# Motivations for Self-Harm in Young People and Their Correlates: A Systematic Review

**DOI:** 10.1007/s10567-024-00511-5

**Published:** 2025-01-29

**Authors:** S. Tang, A. Hoye, A. Slade, B. Tang, G. Holmes, H. Fujimoto, W.-Y. Zheng, S. Ravindra, H. Christensen, A. L. Calear

**Affiliations:** 1 Black Dog Institute, UNSW Sydney, Sydney, NSW Australia; 2 Centre for Mental Health Research, Research School of Population Health, Australian National University, Canberra, ACT Australia; 3 Faculty of Medicine and Health, UNSW Sydney, Sydney, NSW Australia

**Keywords:** Self-harm, Non-suicidal self-injury, Young people, Motivations

## Abstract

Self-harm in young people is associated with increased risk of suicide and other negative long-term outcomes. Understanding the motivations driving self-harm behaviours among young people can help to inform the development of preventative and treatment interventions. Self-harm rates have been rising, but reviews of the recent quantitative literature have not been undertaken. PsycInfo, Embase and Medline were systematically searched in September 2024 for studies published in the past ten years. Quantitative studies that examined motivations for self-harm (including prevalence and/or correlates) among young people (aged 10 to 24 years) with a history of self-harm were included in the review. The review was conducted in accordance with PRISMA guidelines and registered with PROSPERO (CRD42023429568). One hundred and seventeen studies met inclusion criteria. Intrapersonal motivations for self-harm (particularly emotion regulation, anti-dissociation and self-punishment) were more common than interpersonal motivations (e.g. peer bonding, communication). Intrapersonal motivations correlated with female gender, higher self-harm severity, current, repetitive and persistent self-harm, suicidality, poorer mental health and poorer emotion regulation. There was evidence to suggest that interpersonal motives are associated with younger age and some mental health difficulties (e.g. anxiety). Young people predominantly self-harm for intrapersonal reasons. Given that self-harm for intrapersonal reasons is associated with greater self-harm severity, suicidality and poor mental health, steps should be taken to prevent and reduce self-harm. Interventions for self-harm require a multifaceted approach that not only provides young people with alternate ways of regulating their emotions, but also targets risk factors that contribute to self-harm.

**Supplementary Information:**

The online version contains supplementary material available at 10.1007/s10567-024-00511-5.

## Introduction

Self-harm involves intentionally causing pain or damage to one’s body, with or without suicidal intent (Hawton & Fortune, 2008; Mindframe, 2024). Internationally, rates of self-harm among young people have increased considerably over the past decade, particularly among young women. In Australia, for example, rates of self-harm hospitalisations among females aged 15–19 years have almost doubled from 374 to 637 hospitalisations per 100,000 over the ten-year period between 2008–2009 and 2020–2021 (Australian Institute of Health & Welfare, 2023). Evidence from the National Survey of Mental Health and Wellbeing indicates that the rate of self-harm not resulting in a hospital admission may be up to ten times higher (Australian Bureau of Statistics, 2020–2022). Similar trends have been identified across other high-income countries, including the US, UK, Ireland and Canada (Cybulski et al., 2021; Gardner et al., 2019; Griffin et al., 2018; Mercado et al., 2017).

The increasing rate of self-harm among young people is concerning, particularly given that self-harm is associated with increased risk of suicide, depression, anxiety, illicit substance use/dependence and poorer educational and employment outcomes (Borschmann et al., 2017; Duarte et al., 2020a, 2020b; Hawton et al., 2012; Mars et al., 2014). Given the increasing rates of self-harm and poor outcomes associated with self-harm, it is essential that we seek to better understand the contemporary drivers of self-harm behaviours among young people, including precipitants and motivations for self-harm. This knowledge could assist in the development of detailed theoretical models, and the subsequent development of intervention programmes.

Multiple instruments have been used to measure motivations for self-harm, such as the Inventory of Statements About Self-Injury (ISAS; Klonsky & Glenn, 2009) and the Functional Assessment of Self-Mutilation (FASM; Nock & Prinstein, 2004, 2005). These instruments have typically been used to assess motivations for non-suicidal self-injury (NSSI)—that is, self-harm without suicidal intent. Factor analyses using these tools have generally revealed a two-factor solution to the functions assessed by these scales: intrapersonal motivations and interpersonal motivations (Kortge et al., 2013). Intrapersonal (or “automatic”) motivations for self-harm are motivations that are self-focussed and relate to how the act of self-harm can reinforce itself (e.g. emotion regulation, escape from suicidal thoughts, escape from dissociative feelings, self-punishment), while interpersonal (or “social”) motivations for self-harm are motivations that are socially reinforced (e.g. peer bonding, communicating pain to others, sensation-seeking, revenge).

The four-function model of NSSI further divides these two motivations by positive and negative reinforcement (Bentley et al., 2014). Intrapersonal/Automatic motivations can be categorised into automatic negative reinforcement, which occurs when self-harm reduces or provides escape from unwanted emotions or thoughts, or automatic positive reinforcement, which refers to when the sensations that arise as a result of self-harm reinforce this behaviour (e.g. feeling satisfaction). Interpersonal/Social motivations can be divided into social negative reinforcement, when self-harm facilitates escape from social situations or reduces interpersonal demands, or social positive reinforcement, when self-harm facilitates positive social outcomes (e.g. access to resources, help or attention from others).

Existing reviews have consistently found that intrapersonal motivations are the most common motivations for self-harm (Klonsky, 2007). However, self-harm is likely to serve multiple functions for those engaging in this behaviour (Klonsky, 2007), and there are individual differences in why people self-harm (Coppersmith et al., 2021; Shahwan et al., 2020; Singhal et al., 2021). Indeed, studies have shown that self-harm motivations can differ by gender, age and country (Mannekote Thippaiah et al., 2021; Troya et al., 2019). For instance, a study by Gandhi et al. (2021) found that intrapersonal motivations are more strongly endorsed among young people from Belgium, compared to those from India. A number of studies have also found that intrapersonal motivations are more strongly endorsed by females compared to males (e.g. Faura-García et al., 2022).

Previous systematic reviews have sought to characterise different motivations for self-harm among young people (e.g. Edmondson et al., 2016; Taylor et al., 2018; Valencia-Agudo et al., 2018). However, these reviews have several limitations. Namely, although existing reviews have examined correlates of self-harm in young people (e.g. Fliege et al., 2009), no reviews to our knowledge have identified correlates of different self-harm motivations. Doing so would allow us to better characterise different subgroups of young people who engage in self-harm. Identifying such subgroups may allow for the development of more extensive theoretical models, and more tailored interventions to reduce self-harm. There has also been a lack of reviews conducted in the past five years, during which time self-harm has increased significantly and self-harm motivations among young people may have evolved (Australian Institute of Health & Welfare, 2023; Sara et al., 2023). The aim of this study was therefore to characterise motivations for self-harm among young people, including the prevalence of different motivations, and to examine their correlates. Given gender differences in rates of self-harm, we were also particularly interested in the relationship between gender and motivations for self-harm.

## Method

### Search Strategy and Selection Criteria

This systematic review adhered to Preferred Reporting Items for Systematic Reviews and Meta-Analyses (PRISMA) guidelines (Page et al., 2021). The protocol was prospectively registered with PROSPERO (registration number CRD42023429568). However, given that the volume and breadth of included studies exceeded our initial expectations, we chose to present the results of the registered review across multiple articles. This paper examines the motivations for self-harm. A second paper will examine precipitants of self-harm (i.e. stressors immediately preceding self-harm, such as relationship difficulties and school problems) among young people, including their frequency and subgroup differences. A third paper will synthesise qualitative studies, focussing on both motivations and precipitants of self-harm. The latter paper requires thematic analysis and extraction using qualitative methods, which justifies the need for a separate paper.

Three electronic databases (PsycInfo, Embase & Medline) were searched using three key blocks of terms related to i) young people, ii) self-harm and suicide and iii) motivations and functions (see Online Resource 1 for the search strategy used in each database). Publication date was restricted to the past 10 years, with the original search having taken place on 23rd March 2023. An updated search was subsequently conducted on 10th September 2024. No restrictions were placed on language. Reference lists of included studies and relevant past reviews were subsequently examined to identify any additional papers.

## Eligibility Criteria

Eligible studies examined motivations for self-harm among young people (including prevalence and/or correlates) and met all the following requirements:

(i) *Study design.* We included quantitative studies, including cross-sectional, longitudinal and case series designs, that were published in a peer-reviewed journal. Qualitative studies, systematic reviews, meta-analyses, case studies, conference abstracts and book chapters were excluded from this review. However, qualitative studies will be thematically synthesised in a separate review.
(ii) *Self-harm definition.* We defined self-harm as an act with a non-fatal outcome in which an individual deliberately initiates behaviour or ingests an illicit drug or medication or non-ingestible substance or object, with the intention of causing harm to themselves. While we did not seek to distinguish between self-harm with and without suicidal intent, we excluded studies that were specifically focussed solely on young people engaging in a suicide attempt. This is because we were interested in motivations outside of suicide.
(iii) *Population of interest.* We included young people, aged 10 to 24 years (as defined by the World Health Organisation), with a history of self-harm. Studies were included if the participant age range or mean age fell within 10–24 years. Studies that examined multiple age groups were included if they conducted analyses of interest exclusively among young people.
(iv) *Year of publication.* The original search included papers published in the ten years up until 23rd March 2023. An updated search was conducted on the 10th September 2024 to identify additional articles published since the initial search. We restricted the search to the past 10 years given the significant increase in rates of self-harm among young people during this time period. We were specifically interested in better understanding contemporary motivations for self-harm and their correlates, so as to provide insight into factors driving recent self-harm trends.
(v) *Language.* We only included papers that were published in the English language.

## Study Selection

**Fig. 1:**
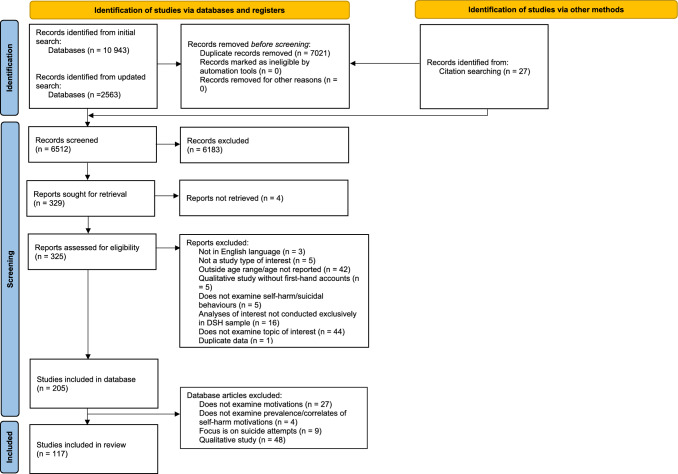
PRISMA flow diagram

A flow chart of study identification and selection is presented in Fig. 1. Following the removal of duplicates, all titles/abstracts were independently double-screened by ST, HF, AH, AS or WZ using Covidence (Covidence, 2021). Average agreement between authors was 93.92% (mean κ = 0.53). Disagreements were resolved through discussion. All full-text articles were double-screened by ST and HF, AH, AS or WZ, with disagreement resolved through discussion. Average agreement between authors was 90.55% (mean κ = 0.80).

### Data Extraction and Synthesis

For each included study, data were extracted independently by two authors (ST, AH, AS, BT or GH) using Covidence. Extracted data for all included studies were compared and checked by ST via Covidence, and disagreements were resolved through discussion. The following data were extracted for each study: bibliographic information (author, year), country, study design, sample size, participant description, participant characteristics (age, gender, representativeness), measures used, self-harm method examined (specific or mixed) and key findings.

The diversity of study methodologies, analyses and correlates examined by each study precluded a meta-analytic approach to the synthesis of data. For example, studies examining the prevalence of different self-harm motivations differed in the type of measure used, in whether they reported on mean scale scores or the percentage of participants endorsing a particular motivation, and on whether they reported on the prevalence of intrapersonal and interpersonal motivations as composites, or whether they reported on the prevalence of specific types of intrapersonal and interpersonal motivations. As such, a narrative synthesis approach was used. We initially summarised studies comparing the prevalence of different types of self-harm motivations. We then summarised correlates of different self-harm motivations. Results were grouped according to correlate type (e.g. gender, age, nature of self-harm, mental health conditions). Only correlates that were examined in multiple papers were summarised in the results section.

### Quality Assessment

The quality of included studies was assessed using a modified version of the Joanna Briggs Institute (JBI) Checklists for Cohort and Analytical Cross-Sectional Studies (Munn et al., 2014; see Online Resource 2). A similar adaptation of this tool was used in a previous systematic review by our team (Tang et al., 2022), as well as a past systematic review of self-harm motivations in young people (Taylor et al., 2018). Five items were selected and modified based on their relevance for the current review: (1) Were the criteria for inclusion in the sample clearly defined? (2) Were the study subjects and the setting described in detail? (3) Was the exposure (self-harm) measured in a valid and reliable way? (4) Was the outcome (i.e. function/motivation of self-harm) measured in a valid and reliable way? (5) Was appropriate statistical analysis used? Studies were rated as ‘adequate’, ‘partial’, ‘poor/unclear’ for each item. Quality assessment was performed independently by two authors (ST, AH, AS, BT or GH). Quality assessment ratings for all studies were checked by ST, and disagreements were resolved through discussion.

## Results

### Study Characteristics

A total of 10 943 articles were identified in the initial search, while a total of 2563 additional articles were identified in the updated search. After removing duplicates and excluding studies based on titles/abstracts, full-text or overlapping samples, 117 were eligible for inclusion in this review (see Fig. 1). Characteristics of included studies are presented in Table 1. Sample sizes ranged from 7 to 13,396. The majority of included studies were conducted in the USA, Canada and Australia. All included studies examined a mix of different self-harm methods, or did not specify the self-harm method they examined. Twenty-seven studies examined prevalence of self-harm motivations only (without also examining correlates of self-harm motivations). Commonly used tools to assess self-harm included the ISAS (*n* = 42), SITBI (*n* = 16) and FASM (*n* = 15). The same tools were frequently used to assess self-harm motivations (ISAS: *n* = 47; FASM: *n* = 21; SITBI: *n* = 10). A total of 29 (24.8%) studies used a representative sample.

**Table 1:** Characteristics of all included studies

Author (year); country	Study design	Sample source	Representative (Y/N)	Sample size (% female)	Age range in years (M, SD)	Self-harm measure(s) (time period)	Motivation measure(s)	Correlates (measure)
Abbasian et al. (2021); Iran	Cross-sectional	High school students	N	604 (100%) NSSI subsample: 160 (100%)	Overall sample: 14–17 (M = 14.3, SD = 1.1) NSSI sample: 14–17 (M = 14.3, SD = 1.1)	ISAS (lifetime)	ISAS	- NSSI severity (ISAS, DSM-5)
Ammerman et al. (2021); USA	Cross-sectional	University students	N	977 (83%)	18–47 (M = 20.1, SD = 2.7)	ISAS (lifetime)	ISAS	- NSSI disclosure (non-validated measures, e.g. “Have you disclosed, or told someone, about your behavior?”)
Andrei et al. (2024); Romania	Case series	Inpatient sample	N	100 (80%)	13–17 (M = 14.9, SD = 1.5)	NSSI-AT –Romanian version (lifetime)	NSSI-AT—Romanian version	- Gender
Andrewes et al. (2017); Australia	EMA	Clinical (BPD) sample	N	107 (83.2%) NSSI subsample: 24 (87.5%)	Overall sample: 15–25 (M = 18.1, SD = 2.7)NSSI sample: 15–25 (M = 18.2, SD = 2.9)	Parasuicidal Behaviours subscale of the BPD-SI (3 months)	Non-validated measure	N/A
Armiento et al. (2014); Canada	Cross-sectional	University students	N	836 (71%) NSSI subsample: 268 (70.3%)	Overall sample: range NR (M = 19.2, SD NR) NSSI sample: M = 19.1 (SD and range NR)	ISAS (lifetime), SBQ-R (12 months)	ISAS	- NSSI disclosure (non-validated measure; “Have you told anyone that you self-harm?”
Babcock Fenerci et al. (2022); USA	Cross-sectional	Inpatient sample Control participants: community	N	124 NSSI subsample: 49 (79.6%)	Overall sample: 13–17 NSSI sample: M = 14.9, SD = 1.3	SITBI (lifetime)	SITBI	- History of maltreatment (CTQ)
Bahali et al. (2024); Turkey	Cross-sectional	Inpatient sample Control participants: community	N	106 (70%) NSSI subsample: 50 (82%)	Overall sample: 14–18 (M = 15.3, SD = 0.9) NSSI subsample: 14–18 (M = 15.6, SD = 1.1)	ISAS (lifetime)	ISAS	- Parental relationships (PBI) - Suicidality (attempts) - Trauma (CTQ-28)
Barreto Carvalho et al. (2017); Portugal	Cross-sectional	High school students	Y	1763 (52.9%) NSSI subsample: 521 (53.2%)	Overall sample: 14–22 (M = 16.8, SD = 1.31) NSSI sample: 14–22 (M = 16.5, SD = 1.2)	ISSIQ-A (lifetime)	ISSIQ-A	N/A
Batejan et al. (2015); USA	Cross-sectional	University students	N	367 (73%) NSSI subsample: 207 (minor NSSI: 67.6%; moderate/severe NSSI: 83.3%)	Overall sample: 17–45 (M = 20.6, SD = 3.1) Minor NSSI sample: 18–37 (M = 20.6, SD = 3.1) Moderate/severe NSSI sample: 18–45 (M = 20.4, SD = 3.4)	ISAS (lifetime)	ISAS	- NSSI severity (ISAS)
Bentley et al. (2014); USA	Cross-sectional	University students	N	150 (71.3%)	18–24 (M = 18.8, SD = 1.0)	ISAS (lifetime)	ISAS	- Experiential avoidance (MEAQ) - Depression (ODSIS) - Anxiety (OASIS)
Braga and Gonçalves (2014); Portugal	Cross-sectional	University students	N	518 (67.0%) NSSI subsample: 84	Overall sample: 17–62 (M = 20.9, SD = 6.1) Self-injurer sample: M = 19.5 (SD and range NR)	SIQ-TR (lifetime)	SIQ-TR	N/A
Brausch et al. (2016); USA	Study 1 = cross-sectional Study 2 = case series	Study 1: University students Study 2: inpatient sample	N	Study 1: 2950 (71.2%) NSSI threshold subsample: 105 NSSI subthreshold subsample: 115 Study 2: 1082 (88.1%) NSSI threshold subsample: 539 NSSI subthreshold subsample: 543	Study 1: Overall sample: Range NR (M = 20.4, SD = 3.0) Study 2: Overall sample: 12–57 (M = 17.4, SD = 6.7) Age of NSSI subsamples NR	Study 1: SITBI—short form (lifetime) and ISAS (lifetime) Study 2: ABASI (past year) & ISAS (lifetime)	ISAS	- NSSI frequency (SITBI, ISAS, ABASI)
Brausch and Muehlenkamp (2018); USA	Cross-sectional	University students	N	264 (84%)	18–35 (M = 19.1, SD = 1.8)	SITBI (lifetime)	Modified ISAS Section II	- Effectiveness of NSSI in achieving function (modified ISAS) - NSSI versatility (SITBI) - NSSI frequency (SITBI) - Suicidality (ideation, plans, attempts; SITBI)
Calvete et al. (2015); Spain	Cross-sectional	High school and vocational school students	N	1864 (51.4%) NSSI subsample: 999 (58%)	Overall sample: 12–19 (M = 15.3, SD = 2.0) Age of NSSI subsample NR	FASM (past year)	FASM	- Gender (demographic questionnaire)
Carranza et al. (2022); Canada	Cross-sectional	University students	N	1018 (70%) NSSI subsample: 246	Range NR (M = 19.8, SD = 3.0) Age of NSSI subsample NR	ISAS (lifetime)	ISAS	- Presence of interpersonal violence (CTS2)
Carvalho et al. (2023a); Portugal	Cross-sectional	High school students	Y	7918 (53.3%) NSSI subsample: 1815 (56.4%)	Overall sample: 13–19 (M = 15.5, SD = 1.7) Age of NSSI subsample NR	ISSIQ-A (timeframe NR)	ISSIQ-A	- Age - Early memories of warmth and safeness (EMWSS-A) -—Gender
Carvalho et al. (2023b); Portugal	Cross-sectional	High school students	Y	7918 (53.3%) NSSI subsample: 1815 (56.4%)	Overall sample: 13–19 (M = 15.5, SD = 1.7) Age of NSSI subsample NR	ISSIQ-A (timeframe NR)	ISSIQ-A	- Parental care (CECA-Q) - Emotion regulation - Trauma (CECA-Q)
Case et al. (2020); USA	Cross-sectional	University students	N	359 (NR)	Range NR (M = 20.4, SD = 3.3)	DSHI (lifetime and past year)	ISAS	- NSSI severity (DSHI)
Christoforou et al. (2021); Australia	Cross-sectional	University students	N	270 (84.1%)	17–56 (M = 21.5, SD = 5.3)	ISAS (lifetime)	ISAS	- Emotion difficulties (DERS)
Coppersmith et al. (2021); USA	EMA (aggregated data from 3 previous EMA studies)	Study 1: Outpatient sample Study 2: Community and online	N	Study 1: 7 (86%) Study 2: 15 (93%) Study 3 outside age range of interest	Study 1: M = 22 (SD and range NR) Study 2: M = 17 (SD and range of final sample NR; only initial criterion ‘12–19 years old’ is reported—prior to excluding participants)	EMA (daily)	Non-validated measure	N/A
Costa et al. (2021); Brazil	Cross-sectional	Community	Y	505 (49.7%) B-NSSI subsample: 196 (53.1%) n (D-NSSI) = 33 (72.7%)	Overall sample: 12–17 (M = 14.3, SD = 1.6) Age of NSSI subsample NR	FASM—Brazilian version (past year)	FASM—Brazilian version	- Presence of NSSI Disorder diagnosis (DSM-5 diagnostic criteria, FASM Brazilian version)
Czyz et al. (2019); USA	Longitudinal/cohort study	Inpatient sample	N	34 (76.5%) Lifetime NSSI subsample: 29 NSSI during study period: 15	13–17 (M = 15.5, SD = 1.4) Age of NSSI subsample NR	SITBI (lifetime) & C-SSRS (lifetime)	Non-validated measure	- Suicidality (ideation; C-SSRS)
Czyz et al. (2021); USA	EMA (daily diary)	Inpatient sample	N	78 (67.9%)	13–17 (M = 15.2, SD = 1.4)	SITBI (lifetime), adapted ISAS (lifetime)	SITBI	- Suicidality (ideation; C-SSRS, measured during EMA period)
DiCorcia et al. (2017); USA	Cross-sectional	Inpatient sample	N	263 (59%) NSSI subsample: 193 (60%)	13–25 (M = 18, SD = 3.5)	Adapted SITBI (lifetime)	Adapted SITBI	- Age - Anxiety (PHQ-4) - Depression (PHQ-4) - Gender - Suicidality (ideation, attempts; C-SSRS)
Dixon-Gordon et al. (2022); Sample 1: Global (majority USA, Canada, UK & Australia) Sample 2: USA & Canada	Cross-sectional	Sample 1: Online Sample 2: Community	N	Sample 1: 155 (96.1%) Sample 2: 127 (82.7%)	Sample 1: 15–35 (M = 21.2, SD = 4.2) Sample 2: 18–35 (M = 23.8, SD = 4.9)	Sample 1: QNSSI (lifetime, past 3 months) Sample 2: DSHI (lifetime)	Sample 1 & 2: QNSSI, SASII	N/A
Doyle (2017); Ireland	Cross-sectional	Post-primary school students	Y	856 (48.8%) NSSI subsample: 103	15–17 (mode = 16; M and SD NR)	Lifestyle and Coping survey (lifetime)	Lifestyle and Coping survey	N/A
Doyle et al. (2017); Ireland	Mixed methods	High school students	Y	856 (48.8%) NSSI subsample: 103 (72.8%)	15–17 (M and SD NR)	Lifestyle and Coping survey (lifetime)	Lifestyle and Coping survey	N/A
Duarte et al. (2019); Portugal	Cross-sectional	High school students	N	411 (53.3%) Self-harm subsample: 109	Overall: 12–19 (M = 15, SD = 1.9) Age of NSSI subsample NR	Inventory of Deliberate Self-Harm Behaviours (adapted from ISAS; lifetime)	Questionnaire of Representations About the Functions of Deliberate Self-Harm for Adolescents (adapted from ISAS)	N/A
Duarte et al., (2020a, 2020b); Portugal	Cross-sectional	High school students	N	203 (54.2%) NSSI subsample: 51	Overall adolescent sample: 12–19 (M = 14.7, SD = 1.8) Age of NSSI subsample NR	Inventory of Deliberate Self-Harm Behaviours (lifetime)	Questionnaire of Representations about the Functions of Deliberate Self-Harm	N/A
Faura-Garcia et al. (2022); Spain	Cross-sectional	Secondary and/or high school students	N	685 (60.9%) NSSI subsample: 115 (72.2%)	Overall sample: 13–18 (M = 15.9, SD = 1.1) Age of NSSI subsample NR	SITBI (lifetime)	SITBI, FASM	- Gender
Gandhi et al. (2016); Belgium	Cross-sectional	High school students	N	401 (51.1%) NSSI subsample: 66 (65.2%)	Overall: 14–19 (M = 16.6, SD = 1.0) Age of NSSI subsample NR	Modified SIQ-TR (lifetime)	Modified SIQ-TR	- Autonomy/identity (PSI-II)
Gandhi et al. (2021); India & Belgium	Cross-sectional	Indian sample: University students Belgian sample: Existing datasets	N	276 (57.2%) NSSI subsample: 55	Indian: Range NR (M = 19.8, SD = 3.2) Belgium: Range NR (M = 20.3, SD = 2.9) Age of NSSI subsample NR	Non-validated measure (lifetime)	Adapted SIQ-TR	- Country (Indian vs. Belgian)
García-Nieto et al. (2015); Spain	Cross-sectional	Outpatient sample	N	267 (34.7%) NSSI subsample: 58 (38%)	Overall sample: 11–18 (M = 14.1, SD = 1.9) NSSI: 11–18 (M = 14.3, SD = 1.8)	SITBI—Spanish version (lifetime)	SITBI—Spanish version	N/A
Gardner et al. (2021); UK	Longitudinal/cohort study	Avon Longitudinal Study of Parents and Children	N	528 (79.9%)	Examined functions of self-harm at age 16 and 21 Repeat self-harm examined at ages 21, 24 & 25	Non-validated measure (lifetime)	Non-validated measure	- Age - Self-harm repetition (past year frequency at follow-up) - Future suicide attempts (suicide attempts at follow-up)
Gatta et al. (2022); Italy	Retrospective cohort study	Inpatient sample	N	361 (69.8%) NSSI subsample: 147	Overall sample: 1–18 (M = 13.4, SD = 3.0) NSSI sample: range NR (M = 14.8, SD = 1.2)	Hospital/clinical records (lifetime)	Hospital/clinical records	N/A
Gholamrezaei et al. (2023); Iran	Cross-sectional	University students	N	63 (52.4%)	Range NR (M = 22.2, SD = 2.8)	One bespoke question (lifetime)	Bespoke questionnaire adapted from HIDS, ISAS	- Anxiety - Depression (DASS-21) - NSSI characteristics (including emotions experienced after NSSI engagement, age of onset, severity, presence of pain, aloneness while engaging in NSSI; adapted HIDS and ISAS) - Gender - Suicidality (SBQ-R)
Goddard et al. (2021); Australia	Cross-sectional	University students	N	236 (83%)	17–56 (M = 21.6, SD = 5.4)	ISAS (lifetime)	ISAS	- Personality traits (BFI-44)
Gray et al. (2022); Australia	Cross-sectional	University students	N	374 (80.7%) NSSI subsample: 210	Overall sample: 18–52 (M = 23.6, SD = 4.2) Age of NSSI subsample NR	ISAS (lifetime)	ISAS	- NSSI status (current or stopped; ISAS) - Desire to stop NSSI (ISAS)
Groschwitz et al. (2015); Germany	Cross-sectional	Inpatient sample	N	111 (65.8%) NSSI disorder subsample: 41 SBD subsample: 34 (82.4%)	Overall sample: 12 -19 (M = 15.4, SD = 1.7) SBD subsample: 12–19 (M = 15.4, SD = 1.5) Age of NSSI subsample NR	SITBI-G (German version) (lifetime)	SITBI-G	N/A
Guan et al. (2024); China	Cross-sectional	Outpatient sample	N	412 (70.2%)	13–18 (M = 15.0, SD = 1.6)	NSSI diagnosis by psychiatrist (timeframe NR)	ANSSIAQ	N/A
Guérin-Marion et al. (2021); Canada	Cross-sectional	University students	N	479 (83.8%)	17–25 (M = 18.8, SD = 1.4)	OSI (past year, lifetime)	OSI	- Emotion regulation difficulties (DERS, DERS-positive, RTSQ)
Güngördü and Ayaydin (2024); Turkey	Case-controlled cross-sectional study	Outpatient sample	N	110 (68.2%) NSSI subsample: 50 (70%)	Overall sample: 13–17 (M = 15.5, SD = 1.3) NSSI subsample: 13–17 (M = 15.4, SD = 1.4)	ISAS (lifetime)	ISAS	- Gender
Hamza et al. (2014); Canada	Experimental study	University students	N	82 (69.5%)	M = 21.5 (SD & range NR)	ISAS (past year)	ISAS	- Pain tolerance (cold-pressor task)
Hettiarachchi et al. (2018); Sri Lanka	Cross-sectional	Justice-involved youth	Y	181 (28.2%) Self-harm subsample: 77 (42.9%)	Overall sample: 12–16 (M = 15.0, SD = 2) Age of NSSI subsample NR	Non-validated measure (lifetime)	Non-validated measure	N/A
Horowitz and Stermac (2018); Canada	EMA	Community	N	38 (89.5%)	18–30 (M = 21.9, SD = 2.6)	ISAS (lifetime)	ISAS	- Trauma severity (TEC)
Idig-Camuroglu and Gölge (2018); Turkey	Cross-sectional	University students	N	1000 (69%) NSSI subsample: 285 (65.3%)	Range, M & SD NR Overall sample: 46.1% between 18–20 years, 45.1% 21–23 years old, 8.8% 24 and older	ISAS (lifetime)	ISAS	- Gender - Trauma (CTQ)
Ilieff and Hamza (2023); Canada	Longitudinal	University students	N	841 (71%) NSSI subsample time 1: 225 (NR) NSSI subsample time 2: 185 (NR) NSSI subsample time 3: 126 (NR)	Overall sample: 18–25 (M = 18.0, SD = 0.7) NSSI subsample: range NR (M = 18.0, SD = 0.8)	Adapted ISAS (past 4 months)	Adapted ISAS	- Trauma (PCL)
Jiang et al. (2022); China	Cross-sectional	Junior high school students	N	2376 (52.2%) NSSI subsample: 881 (56.6%)	Overall sample: range NR (M = 13.7, SD = 1.0) NSSI: range NR (M = 13.6, SD = 1.0)	Adolescents Self-Harm Scale (past year)	Adolescents Self-Harm Scale	N/A
Jonsson et al. (2019); Sweden	Cross-sectional	High school students	Y	NSSI only subsample: 910 (76%) SASI only subsample: 41 (70.7%) NSSI & SASI subsample: 76 (86.8%)	Overall sample: range NR (M = 18.0, SD = 0.6) Age of NSSI subsamples NR	SITBI-SF-SR (lifetime)	FASM	- Self-injury method (sex as a form of self-injury vs. other NSSI methods) (FASM & non-validated measure: “Have you ever used sex to purposely hurt yourself?”)
Kaess et al. (2013); Germany	Case series	Inpatient sample	Y	125 (50.4%) NSSI subsample: 75 (57.3%)	Overall: 13–26 (M = 17.1, SD = 3.1) NSSI: 13–26 (M = 16.5, SD = 2.6)	FASM (past year)	FASM	- Adverse childhood experiences (CECA-Q)
Kandsperger et al. (2022); Germany	Longitudinal/cohort study	Outpatient sample	Y	97 (77.3%) NSSI subsample: 88	11–18 (M = 14.9, SD = 1.52)	SITBI-G (lifetime)	SITBI-G	- Emotional reactivity (ERS)
Ke et al. (2024); China	Cross-sectional	High school students	N	3657 (44.2%) NSSI subsample: 616	Overall sample: 13–20 (M = 16.5, SD = 1.1) NSSI subsample: 13–20 (M = 16.6, SD = 1.1)	ANSAQ (past year)	ANSAQ	- Alexithymia (TAS-20) - Childhood maltreatment (CTQ-SF) - Help-seeking attitudes (ATSPPH-SF)
Kharsati and Bhola (2015); India	Cross-sectional	University students	N	470 (70%) NSSI subsample: 143	Overall sample: range NR (M = 20.3, SD = 1.7)	FASM (past year)	FASM	- Severity of NSSI (FASM)
Khutoryanskaya et al. (2023); Russia	Case series	Outpatient sample	N	91 (87.9%)	13–24 (M = 17, SD NR)	ISAS – Russian version (lifetime)	ISAS – Russian version	- Age - Gender - Mental health diagnoses (including behavioural disorders, depressive episodes and eating disorders; clinical assessment by a psychiatrist)
Kiekens et al. (2017); USA	Longitudinal/cohort study	College students	Y	Persistent NSSI subsample: 51 (67.3%) Ceased NSSI subsample: 50 (83.7%)	Persistent NSSI: range NR (M = 20, SD = 3.0) Ceased NSSI: range NR (M = 20.3, SD = 2.4)	NSSI-AT (lifetime)	NSSI-AT	- NSSI status (ceased vs. Persistent; NSSI-AT at baseline and follow-up)
Klonsky et al. (2015); USA	Case series	Inpatient sample	N	1157 (89.4%)	11–73 (M = 16.6, SD = 7.7)	ISAS (lifetime), FASM (past year)	ISAS, FASM	- Recent NSI frequency (ABUSI) - Recent NSI urge (ABUSI)
Knorr et al. (2013); USA	Cross-sectional	University students	N	1678 (70.5%) NSSI subsample: 359 (75.5%)	Total N: 18–27 (M = 20.4, SD = 1.6) NSSI: 18–27 (M = 20.3, SD = 1.6)	FAFSI (lifetime)	FAFSI	- Total number of NSI acts (FAFSI) - NSSI types (FAFSI) - NSSI method (FAFSI) - Sensation seeking (SSPT) - Emotional reactivity (ERS)
Kostić et al. (2019); Serbia	Case series	Clinical sample	N	50 (70%)	13–18 (M = 15, SD = 1.2)	ISAS (lifetime)	ISAS	- Gender
Kostic et al. (2024); Serbia	Case series	Inpatient sample	N	50 (82%)	12–18 (M = 15.4, SD = 1.4)	Hospital records (timeframe NR)	OSI	- Motivation to stop NSSI (OSI) - Suicidality (ideation; OSI)
Kraus et al. (2020); Switzerland & Germany	Cross-sectional	Inpatient sample	N	56 (100%)	12–18 (M = 16.0, SD = 1.3)	Kinder-DIPS (lifetime), FASM (past year)	Kinder-DIPS, FASM	- Suicidality (ideation; Kinder-DIPS)
Lee (2016); South Korea	Cross-sectional	Middle school students	N	784 (48.8%) NSSI subsample: 97	Overall sample: 13–15 (M = 14.4, SD = 1.7) Age of NSSI subsample NR	SHQ (lifetime)	SHQ	N/A
Luo et al. (2024); China	Cross-sectional	Primary and middle school students	N	10,501 (49.5%) NSSI subsample (NSSI 5 + times in past year): 697 (61.3%)	Overall sample: 8–18 (M & SD NR) Age of NSSI subsample NR	OSI (past year)	OSI	- Addictive features of NSSI (OSI) - Severity of NSSI (ASHS)
Luyckx et al. (2015); Belgium	Cross-sectional	Sample 1: High school students Sample 2: Inpatient sample	Y	Sample 1: 348 (100%) NSSI subsample: 72	Overall: range NR (M = 16.0, SD = 1.3) Age of NSSI subsample NR	SIQ-TR (lifetime)	SIQ-TR	- Identity (EPSI Identity subscale)
Ma and Su (2023); China	Cross-sectional	Junior high school students	N	1885 NSSI subsample: 544	Overall sample: 11–16 (M = 13.1, SD = 0.9) NSSI subsample: 11–16 (M = 13.1, SD = 0.9)	OSI (past year)	OSI	- Gender
Mahtani et al. (2018); Australia	Cross-sectional	University students	N	384 (81.3%)	Range NR (M = 20.7, SD = 2.3)	ISAS (lifetime)	ISAS	N/A
McManus et al. (2019); England, UK	Longitudinal/cohort study	Community	Y	Year 2000: 665 (NSSI subsample: 37) Year 2007: 567 (NSSI subsample: 59) Year 2014: 559 (NSSI subsample: 90) % female NR	16–24 (M and SD NR)	Non-validated measure (lifetime)	Non-validated measure	- Gender
Mehmood et al. (2023); Pakistan	Cross-sectional	University students	N	386 (55.9%) NSSI subsample: 110 (45.4%)	Overall sample: 17–27 (M = 19.7, SD = 1.5) Age of NSSI subsample NR	ISAS (lifetime)	ISAS	N/A
Mirichlis et al. (2022); Australia	Cross-sectional	University students	N	573 (80.6%)	17–52 (M = 23.7, SD = 6.6)	ISAS (past year)	ISAS	- NSSI disclosure (non-validated measure)
Muehlenkamp et al. (2013); USA	Cross-sectional	University students	N	1243 (NR) NSSI subsample: 183 (NR)	Overall: Range NR (M = 21.5, SD = 4.2) Age of NSSI subsample NR	NSSI-AT (lifetime)	NSSI-AT	- NSSI status (initiating vs. repeating; non-validated measure)
Nagy and Muehlenkamp (2024); USA	Cross-sectional	University students	N	468 (83.4%)	Range NR (M = 21.2, SD = 3.1)	ISAS (lifetime)	ISAS	- NSSI severity (ISAS) - Self-esteem (B-RSE)
Nicol et al. (2022); Australia	Cross-sectional	University and high school students	N	125 (80%)	16–25 University students – female: M = 20.9, SD = 2.4; university students – male: M = 21.4, SD = 2.9; High school students – female: M = 16.4, SD = 0.6; high school students – male: M = 17.1, SD = 0.7	ISAS (lifetime)	ISAS	- Age - Early maladaptive schemas (YSQ)
Ong et al. (2017); Singapore	Cross-sectional	Clinical sample	N	30 (60%)	Range NR (M = 16.3, SD = 1.7)	FASM (past year)	FASM	- Depression (CBCL, psychiatrist interview with diagnoses based on DSM-IV-TR)
Park et al. (2022); Korea	Cross-sectional	Community, online	N	614 (70.8%) NSSI subsample: 414 (NR)	NSSI subsample: 18–29 (M = 22.6, SD = 2.7)	ISAS—Korean version (lifetime)	ISAS	- Suicidality (ideation; BSSI)
Park et al. (2024); USA	Cross-sectional	University students	N	820 (83.3%)	Range NR (M = 20.5, SD = 9.1)	ISAS (lifetime)	ISAS	- Gender - Suicidality (SBQ-R)
Paul et al. (2015); USA	Cross-sectional	University students	Y	13 396 (57.1%) NSSI subsample: 1873	18–29 (M = 20.9–21.8, SD = 2.5–2.8) (M & SD reported separately for different groups)	NSSI-AT (lifetime)	NSSI-AT	- Suicidality (ideation, plans, attempts; non-validated screening questions)
Pérez Rodríguez et al. (2021); Spain	Cross-sectional	Lower secondary, high school, and university students	Y	1733 (53.3%) NSSI subsample: 431	Overall: 12–19 (M = 15.8, SD = 1.8) NSSI: 12–19 (M = 14.9, SD = 1.7)	ISAS – Spanish version (lifetime)	ISAS	N/A
Peters et al. (2019); USA	Baseline measures from an RCT	Inpatient sample	N	52 (62%) NSSI subsample: 49	12–18 (M = 15.6, SD = 1.5) Age of NSSI subsample NR	C-SSRS (lifetime), ISAS (lifetime)	ISAS	- Sexuality (demographic questionnaire)
Piarulli et al. (2023); Italy	Case series	Clinical sample	N	43 (100%)	Range NR (M = 15.2, SD = 1.7)	OSI – Italian version (past year)	OSI – Italian version	- Cortisol levels (serum) - Cortisol/DHEA-S ratio (serum)
Pollak et al. (2020); USA	Case series	Inpatient sample	N	76 (72.3%)	11–17 (M = 15, SD = 1.4)	SITBI (lifetime)	SITBI	- NSSI thoughts and behaviours during and after inpatient admission (SITBI)
Radziwiłłowicz and Lewandowska (2017); Poland	Cross-sectional	Inpatient sample	N	60 (85%)	13–17 (M = 15.5, SD = 1.2)	ISAS – Polish version (lifetime)	ISAS	- Body image (Feelings Towards the Body Questionnaire) - Death of close person (non-validated measure) - Depression (CDI) - Dissociation (Ego-Psychopathology Scale) - Sexual abuse (non-validated measure) - Sexual violence (non-validated measure) - Suicide attempt in family (non-validated measure)
Rasmussen et al. (2016); Northern Ireland, UK	Longitudinal/cohort study	School students	Y	987 (57%) Self-harm subsample: 88 (75%)	Overall: 14–16 (M = 14.7, SD = .6) Age of self-harm subsample NR	Northern Ireland Lifestyle and Coping Survey (lifetime)	Northern Ireland Lifestyle and Coping Survey	- Gender - Repeat self-harm (non-validated measure)
Reinhardt et al. (2021a); Hungary	Cross-sectional	Justice-involved youth	Y	244 (7.4%) Lifetime NSSI subsample: 84 Past month NSSI subsample: 64	Overall: 14–20 (M = 17.0, SD = 1.3) Age of NSSI subsample NR	SIQ-TR (lifetime)	SIQ-TR	- Age - Gender
Reinhardt et al. (2021b); Hungary	Cross-sectional	Secondary school students	Y	1015 (66.1%) NSSI subsample: 418 (70.6%)	Total sample: 14–20 (M = 16.8, SD = 1.4) NSSI (female): M = 16.8, SD = 1.4 NSSI (male): M = 17.0, SD = 1.5	ISAS (parts I and II) – Hungarian version (lifetime), frequency question from the SIQ-TR	ISAS	- Age - Experiential avoidance (AFQ-Y8) - Externalising symptoms (SDQ) - Gender - Internalising symptoms (SDQ) - Loneliness (non-validated measure) - Self-critical rumination (SCRS)
Reinhardt et al. (2022a); Hungary	Cross-sectional	Secondary school students	Y	322 (73.2%)	14–20 (M = 16.7, SD = 1.4)	ISAS (lifetime)	ISAS	- Discrepancy (SAPS Discrepancy subscale) - Mental health (A-MHC-SF) - Pain and aloneness during self-harm (ISAS) - Urge for and commitment to self-harm (ISAS)
Reinhardt et al. (2022b); Hungary	Cross-sectional	Outpatient and inpatient samples	Y	158 (83.5%) Lifetime NSSI subsample: 119 (88.2%) Past month NSSI subsample: 54	Overall: 13–21 (M = 16.1, SD = 1.5) Lifetime NSSI: 13–21 (M = 16.1, SD = 1.6)	ISAS (lifetime)	ISAS	- Age - Co-occurring psychiatric disorders (ICD-10 codes) - Externalising symptoms (ICD-10 codes) - Gender - Internalising symptoms (ICD-10 codes) - NSSI methods (ISAS) - NSSI status (current vs. past, repetitiveness; ISAS) - Self-critical rumination (SCRS)
Robillard et al. (2022); Canada	Longitudinal/cohort study	University students	N	513 (78.2%) NSSI sample at each time point ranged from 10–28	Overall sample: range NR (M = 18.0, SD = .8) Age of NSSI subsample NR	Non-validated measure (past month)	24 items adapted from ISAS and SASII	N/A
Robinson et al. (2021); New Zealand	Longitudinal/cohort study	Year 9 students	Y	2057 (54.9%) NSSI subsample: 450	13–18 (M = 15.6, SD = 1.2) Age of NSSI subsample NR	DSHI (lifetime)	ISAS	- Suicidality (ideation and behaviours; SBQ-R)
Rodav et al. (2014); Israel	Cross-sectional	Junior high school and high school students	Y	275 (49.9%) NSSI subsample: 57 (42.1%)	12–17 (M = 14.8, SD = 1.4) NSSI subsample: 12–17 (M = 15.2, SD = 1.2)	OSI-F – Hebrew version (past year)	OSI-F	N/A
Roley-Roberts et al. (2017); USA	Cross-sectional	University students	N	121 (78%)	18–22 (M = 18.7, SD NR)	FASM (past year)	FASM	- Suicidality (ideation, attempts; BSSI)
Sadeh et al. (2014); USA	Case series	Outpatient sample	N	36 (88.6%)	13–24 (M = 16.7, SD = 2.3)	ISAS (lifetime)	ISAS	- BPD symptoms (SCID-II)
Saraff and Pepper (2014); USA	Cross-sectional	University students	N	52 (84.6%)	18–26 (M = 19.8, SD = 1.9)	ISAS (lifetime)	ISAS	- BPD symptoms (SCID-II) - Frequency of NSSI (ISAS) - Status of NSSI (past vs. Recent) (ISAS) - Variety of NSSI (ISAS)
Saraff et al. (2015); USA	Cross-sectional	University students	N	52 (84.6%)	18–26 (M = 19.8, SD = 1.9)	ISAS (lifetime)	ISAS, SASII	- Consequences of NSSI (SASII) - Frequency of NSSI (ISAS)
Schmidt et al. (2023); Spain	Cross-sectional	College students, inpatient sample and online	N	86 (NR) NSSI subsample = 25 (84%) NSSI & BPD subsample = 30 (90%) Healthy controls = 31 (83.9%)	Overall sample: 18–33 NSSI subsample: (M = 20.9, SD = 1.4) NSSI & BPD subsample: (M = 23.6, SD = 4.2) Healthy controls: (M = 23.3, SD = 4.2)	ISAS (lifetime)	ISAS	- Borderline personality disorder diagnosis (SCID-II) - Emotion regulation (DERS-18) - Impulsivity (UPPS-P)
Selby et al. (2014); USA	EMA	Clinical sample	N	30 (86.7%)	12–19 (M = 17.3, SD = 1.9)	Baseline: SITBI (lifetime) EMA: non-validated questions (current)	Non-validated questions asked during EMA	- Age - Dysregulated behaviours (non-validated questions during EMA) - Gender - Impulsiveness of NSSI (non-validated question during EMA) - NSSI frequency (lifetime; SITBI) - Presence of NSSI thoughts/behaviours during EMA (non-validated questions) - Psychiatric disorders (K-SADS)
Shahwan et al. (2018); Singapore	Cross-sectional	Outpatient sample	N	400 (48.8%) Self-harm subsample: 235	14–35 (M = 23.3, SD = 6.0) Age of self-harm subsample NR	FASM (past year)	FASM	N/A
Shen et al. (2023); China	Case series	In- and outpatient sample	N	1101 (81.9%)	12–18 (M = 14.7, SD = 1.6)	C-FASM (past year)	C-FASM	- Age - Gender - NSSI characteristics (including frequency, methods, duration; C-FASM) - Suicidality (past year attempts; non-validated questions)
Shi et al. (2023); China	Cross-sectional	University students	N	1339 (50.3%) NSSI subsample: 116 (50.9%)	Overall sample: 16–29 (M = 19.9, SD = 1.3) NSSI subsample: range NR (M = 19.9, SD = 1.4)	OSI – Chinese version (lifetime)	OSI – Chinese version	- Early maladaptive schemas (YSQ-SF)
Shingleton et al. (2013); USA	EMA	Adolescents who reported engaging in NSSI within the past two weeks	N	30 (87%)	12–19 (M = 17, SD = 1.9)	Baseline: K-SADS (present/lifetime), SITBI (lifetime) EMA: non-validated measures (current)	EMA: non-validated question	N/A
Silverman et al. (2018); USA	Cross-sectional	Justice-involved youth	N	103 (0%) Lifetime NSSI subsample: 68 High-frequency NSSI subsample: 22	13–18 (M = 15.7, SD = 1.1) Age of NSSI subsample NR	DSHI (lifetime)	FDSHA	- Alienation or boredom (APS) - Emotional lability (APS) - Problems with interpersonal relationships (APS)
Szewczuk-Bogusławska et al. (2021); Poland	Mixed method	Inpatient (conduct disorder) sample	N	215 NSSI subsample: 77 (74%)	Lifetime NSSI subsample: range NR (M = 14.6, SD = 1.1)	Non-validated measure (lifetime)	ISAS – Polish version	- NSSI frequency (non-validated measure) - Suicidality (attempts; non-validated measure)
Tan et al. (2014); Singapore	Mixed methods	Outpatient sample	N	60 (60%) NSSI subsample: 30	NSSI: 13–19 (M = 16.3, SD = 1.7) Control: 13–19 (M = 16.0, SD = 1.7)	FASM (past year)	FASM	N/A
Taş Torun et al. (2022); Turkey	Cross-sectional study	Clinical (depression) sample	N	67 (83.6%) NSSI subsample: 43	Girls: 11–17 (M = 14.9, SD = 1.5) Boys: 11–17 (M = 14.7, SD = 1.5) Age of NSSI subsample NR	ISAS (lifetime)	ISAS	- Anxiety (BSI) - Childhood physical abuse (CTQ) - Difficulty identifying feelings (TAS-20) - Emotional neglect (CTQ) - Impulsivity (DERS) - Lack of clarity in emotional response (DERS) - Paranoid ideation (BSI) - Obsessive compulsion (BSI)
Thai et al. (2021); Vietnam	Cross-sectional	High school students	N	1316 (63.3%) NSSI subsample: 551 (61.7%)	Range 15–18 (M & SD NR	FASM (past year)	FASM	- NSSI severity (FASM)
Vega et al. (2017); Spain	Cross-sectional	University students & clinical (BPD) patients	N	274 NSSI subsample: 75 (60.3%) NSSI + BPD subsample: 36 (91.7%)	NSSI-: 18–30 (M = 21.6, SD = 3.5) NSSI + : 18–30 (M = 21.4, SD = 3.2) BPD + NSSI: 18–30 (M = 22.7, SD = 3.5)	ISAS – Spanish version (lifetime)	ISAS	- BPD diagnosis and symptoms (diagnosis based on DSM-IV-TR and DIB-R, symptoms measured using MSI-BPD – Spanish version, BPQ)
Vergara et al. (2023); USA	Case series	Inpatient sample	N	70 (73.6%)	12–17 (M = 14.7, SD = 1.5)	SHBQ (lifetime), ISAS (lifetime)	ISAS	- Suicidality (attempt; SHBQ)
Verroken et al. (2018); Belgium	Cross-sectional	School students (refugee minors aged 14 to 18)	Y	121 (39.7%) Past-year self-harm subsample: 17 Lifetime self-harm subsample: 21 (47.6%)	Total sample: 14–18 (M = 16.1, SD = 1.2)	Screeningsvragenlijst opzettelijk zelfverwondend gedrag (screening questionnaire based on BNSSI-AT) (lifetime)	Screeningsvragenlijst opzettelijk zelfverwondend gedrag	N/A
Victor et al. (2015); USA	Case series	Inpatient, partial hospitalisation and outpatient sample	N	1502 (87.7%)	11–25 (M = 16.4, SD = 2.6)	ABASI (lifetime/past year/past week)	ISAS-SF	- Suicidality (ideation; BASIS-24)
Victor and Klonsky (2018); USA	Cross-sectional	High school students	N	89 (67.4%)	13–17 (M & SD NR)	Non-validated measure (lifetime)	SITBI	- Knowledge of friends’ NSSI (non-validated measure)
Wachter Morris and Wester (2020); USA	Cross-sectional	High school students	N	218 (45.5%) Self-harm subsample: 68 (though analyses of interest only conducted on *n* = 38)	12–18 (M = 15.4, SD = 1.5) Age of self-harm subsample NR	DSHI-A (lifetime)	FASM	- Suicidality (suicidal behaviours; non-validated measures) - Size of peer network (non-validated measure) - Strength of friendships (non-validated measure)
Wang et al. (2022); China	Case series	Outpatient sample	Y	658 (84.4%)	12–18 (M = 15.1, SD = 1.7)	C-FASM (past year)	FASM	N/A
Wang et al. (2024); China	Cross-sectional	College students	N	5281 (67.5%) DiSH only subsample: 488 (78.7%) PSH only subsample: 195 (72.8%) DiSH and PSH subsample: 84 (79.8%)	Range NR Overall sample: (M = 20.8, SD = 2.7) DiSH only subsample: (M = 20.3, SD = 1.8) PSH only subsample: (M = 20.0, SD = 1.8) DiSH and PSH subsample: (M = 20, SD = 1.7)	Bespoke (lifetime)	FASM and bespoke item	- Anxiety (GAD-7) - Depression (PHQ-9) - Method of NSSI (non-validated measure) - Suicidality (ideation, plans, attempts; non-validated measure)
Westers et al. (2014); USA	Cross-sectional	Outpatient sample	Y	30 (70%)	12–19 (M & SD NR)	SITBI – NSSI section (lifetime)	SITBI – NSSI section	- Religious coping (Brief RCOPE) - Religiousness (DRI)
You et al. (2013); Hong Kong	Longitudinal/cohort study	High school students	Y	Wave 1: 5423 (52.7%) NSSI subsample: 1014	Wave 1: 12–18 (M = 14.6, SD = 1.3) Age of NSSI subsample NR	Wave 1: non-validated measure (past year)	Adapted version of FASM	- Gender - NSSI severity (moderate/severe NSSI vs. minor NSSI based on method)
You et al. (2015); Hong Kong	Cross-sectional	High school students	N	42 (76.2%)	14–18 (M = 15.6, SD = 1.1)	ISAS (past 12 months)	SASII	- Self-harm method (SASII)
Zetterqvist et al. (2013); Sweden	Cross-sectional	High school students	Y	3060 (50.5%) NSSI subsample: 1088 (56.2%)	15–17 (M & SD NR)	FASM – Swedish version (past year) & SITBI-SF-SR – Swedish version (lifetime/past year/past month/past week)	FASM	- Gender - NSSI Disorder diagnosis (FASM, SITBI-SF-SR & non-validated diagnostic questions)
Zetterqvist et al. (2014); Sweden	Cross-sectional	Community sample	Y	816 (56.7–57.0%; range is due to missing data on gender)	15–17 (M & SD NR)	FASM – Swedish version (past year)	FASM	- Anxiety (TSCC) - Dissociative symptoms (TSCC) - Emotional abuse (LYLES) - Gender - NSSI Frequency (FASM) - Physical abuse (LYLES) - Prolonged illness or handicap during upbringing (LYLES) - Sexual abuse (LYLES) - Suicide attempt (item from SITBI-SF-SR – Swedish version)
Zhang et al. (2019); China	Cross-sectional	Junior high school students	Y	510 (56.7%)	12–16 (M = 13.5, SD = 1.0)	OSI (past month/past 6 months/past year)	OSI	N/A
Zhao et al. (2024); China	Cross-sectional	Inpatient sample	N	1773 (82.2%)	12–18 (female M = 14.76, SD = 1.65; male M = 15.29, SD = 1.52)	C-FASM (past year)	C-FASM	- Anxiety (GAD-7) - Gender - NSSI frequency (C-FASM)

*ABASI* Alexian Brothers Assessment of Self-Injury, *ABUSI* Alexian Brothers Urge to Self‑Injure Scale, *AFQ-Y8* Avoidance and Fusion Questionnaire for Youth, *A-MHC-SF* Adolescent Mental Health Continuum – Short Form, *ANSAQ* Adolescent Non-suicidal Self-injury Assessment Questionnaire, *ANSSIAQ* Adolescent Non-Suicidal Self-Injury Assessment Questionnaire, *APS* Adolescent Psychopathology Scale, *ASHS* Adolescent Self-Harm Scale, *ATSPPH-SF* Attitudes Towards Seeking Professional Psychological Help Scale–Short Form, *B-RSE* Brief Rosenberg Self-esteem Scale, *B-SCS* Brief Suicide Cognitions Scale, *BASIS-24* Behaviour and Symptom Identification Scale 24, *BFI-44* 44-item Big-Five Inventory, *BNSSI-AT* Brief Non-Suicidal Self-Injury Assessment Tool, *BPD* Borderline Personality Disorder, *BPD-SI* Borderline Personality Disorder Severity Index, *BPQ* Borderline Personality Questionnaire, *Brief RCOPE* Brief measure of Religious Coping, *BSI* Brief Symptom Inventory, *BSSI* Beck Scale for Suicidal Ideation, *C-FASM* Functional Assessment of Self-Mutilation (Chinese Version), *CBCL* Child Behaviour Checklist, *CECA-Q* Childhood Experiences of Care and Abuse Questionnaire, *C-SSRS* Columbia-Suicide Severity Rating Scale, *CTQ* Childhood Trauma Questionnaire, *CTQ-SF* Childhood Trauma Questionnaire Short Form, *CTS2* Revised Conflict Tactics Scale, *DASS-21* The Depression Anxiety Stress Scale, *DERS* Difficulties in Emotion Regulation Scale, *DERS-18* Brief Version of Difficulties in Emotion Regulation Scale, *DERS-positive* Difficulties in Emotion Regulation Scale-Positive, *DHEA-S* Dehydroepiandrosterone sulphate, *DIB-R* Diagnostic Interview for Borderlines-Revised, *DiSH* Digital Self-Harm, *DSHI* Deliberate Self-Harm Inventory, *DSHI-A* Deliberate Self-Harm Inventory Adapted, *DRI* Duke Religion Index, *DSM-5* Diagnostic and Statistical Manual of Mental Disorders, Fifth Edition, *EMA* Ecological Momentary Assessment, *EMWSS-A* Early Memories of Warmth and Safeness Scale – Adolescents, *EPSI* Erikson Psychosocial Stage Inventory, *ERS* Emotion Reactivity Scale, *FAFSI* Form and Function of Self-Injury Scale, *FASM* Functional Assessment of Self-Mutilation, *FDSHA* Functional Deliberate Self-Harm Assessment, *GAD-7* Generalised Anxiety Disorder 7, *EPSI* Erikson Psychosocial Stage Inventory, *HIDS* How I Deal with Stress scale, *ISAS* Inventory of Statements of Self-Injury, *ISSIQ-A* Impulse, Self-harm and Suicide Ideation Questionnaire for Adolescents, *Kinder-DIPS* Diagnostic Interview for Mental Disorders in Children and Adolescents, *K-SADS* Schedule for Affective Disorders and Schizophrenia for School-Aged Children, *LYLES* Linköping Youth Life Experience Scale, *MEAQ* Multidimensional Experiential Avoidance Questionnaire, *MSI-BPD* McLean Screening Instrument for Borderline Personality Disorder, *NR* Not reported, *NSSI* Non-Suicidal Self-Injury, *NSSI-AT* Non-Suicidal Self-Injury Assessment Tool, *OASIS* Overall Anxiety Severity and Impairment Scale, *ODSIS* Overall Depression Severity and Impairment Scale, *OSI* Ottawa Self-Injury Inventory, *OSI-F* Ottawa Self-Injury Inventory-Functions, *PBI* Parental Bonding Instrument, *PCL* Post Traumatic Stress Disorder Checklist, *PHQ-4* Patient Health Questionnaire-4, *PHQ-9* Patient Health Questionnaire-9, *PSH* Physical Self-Harm, *PSI-II* Personal Style Inventory, *QNSSI* Questionnaire for Non-suicidal Self-Injury, *RTSQ* Ruminative Thought Style Questionnaire, *SAPS* Short Almost Perfect Scale, *SASII* Suicide Attempt Self-Injury Interview, *SBD* Suicidal Behaviour Disorder, *SBQ-R* Suicide Behaviour Questionnaire-Revised, *SCID-II* Structured Clinical Interview for DSM-IV Axis II Personality Disorders, *SCRS* Self-Critical Rumination Scale, *SDQ* Strengths and Difficulties Questionnaire, *SHBQ* Self‑Harm Behaviour Questionnaire, *SHQ* Self-harm Questionnaire, *SIQ-TR* The Self-Injury Questionnaire – Treatment Related, *SITBI* Self-Injurious Thoughts and Behaviours Interview, *SITBI-SF-SR* Self-Injurious Thoughts and Behaviours Interview-Short Form-Self Report, *SSPT* Sensation Seeking Personality Type Scale, *TAS-20* Toronto Alexithymia Scale, *TEC* Traumatic Experiences Checklist, *TSCC* Trauma Symptom Checklist for Children, *UPPS-P* Urgency-Premeditation-Perseverance-Sensation Seeking-Positive Urgency Impulsive Behaviour Scale, *YSQ* Young Schema Questionnaire, *YSQ-SF* Young Schema Questionnaire Short Form

### Prevalence of Self-Harm Motivations

Of the 39 studies that compared endorsement of intrapersonal (automatic) motivations for self-harm with endorsement of interpersonal (social) motivations for self-harm, 38 studies found that intrapersonal motivations were more common than interpersonal motivations (Andrei et al., 2024; Barreto Carvalho et al., 2017; Brausch & Muehlenkamp, 2018; Brausch et al., 2016; Carvalho et al., 2023b; Czyz et al., 2021; Dixon-Gordon et al., 2022; Duarte et al., 2019; Gardner et al., 2021; Gatta et al., 2022; Guan et al., 2024; Jiang et al., 2022; Jonsson et al., 2019; Kandsperger et al., 2022; Kharsati & Bhola, 2015; Kostic et al., 2024; Kraus et al., 2020; Mehmood et al., 2023; Muehlenkamp et al., 2013; Ong et al., 2017; Pérez Rodríguez et al., 2021; Peters et al., 2019; Pollak et al., 2020; Rasmussen et al., 2016; Reinhardt et al., 2022b; Robillard et al., 2022; Rodav et al., 2014; Sadeh et al., 2014; Schmidt et al., 2023; Shen et al., 2023; Shi et al., 2023; Tan et al., 2014; Wachter Morris & Wester, 2020; Wang et al., 2022; You et al., 2015; Zetterqvist et al., 2013; Zhang et al., 2019; Zhao et al., 2024). Only one study found that social positive reinforcement motivations (a type of interpersonal motivation) were more common than intrapersonal motivations (Roley-Roberts et al., 2017), Of these 39 studies, 5 studies reported on the percentage of participants who endorsed intrapersonal motivations (as a composite) with that of participants to endorse interpersonal motivations (as a composite). Across these studies, the percentage of participants who endorsed the former ranged from 46.7 to 92.4%, while the percentage of participants who endorsed the latter ranged from 12.6 to 41.8%.

Among studies comparing automatic negative and automatic positive reinforcement functions, automatic negative reinforcement was found to be more common (Braga & Gonçalves, 2014; Coppersmith et al., 2021; García-Nieto et al., 2015; Groschwitz et al., 2015; Kandsperger et al., 2022; Ong et al., 2017; Pollak et al., 2020; Wachter Morris & Wester, 2020; Wang et al., 2022; Westers et al., 2014). This was the case for both suicidal and non-suicidal self-harm (Groschwitz et al., 2015), and for both those who did and did not meet DSM-5 criteria for NSSI disorder (Brausch et al., 2016; Costa et al., 2021; Zetterqvist et al., 2013). Only one study, which utilised an experience sampling methodology, found that automatic positive reinforcement functions were more common compared to automatic negative reinforcement functions (Selby et al., 2014).

A total of 42 studies found that the most common intrapersonal motivations were related to emotion regulation, self-punishment, marking distress and anti-dissociation (Andrewes et al., 2017; Babcock Fenerci et al., 2022; Christoforou et al., 2021; Costa et al., 2021; Czyz et al., 2021; DiCorcia et al., 2017; Doyle et al., 2017; Gandhi et al., 2021; Gholamrezaei et al., 2023; Guan et al., 2024; Güngördü & Ayaydin, 2024; Hettiarachchi et al., 2018; Horowitz & Stermac, 2018; Jiang et al., 2022; Jonsson et al., 2019; Kaess et al., 2013; Kharsati & Bhola, 2015; Khutoryanskaya et al., 2023; Kostić et al., 2019; Lee, 2016; Ma & Su, 2023; Mahtani et al., 2018; Mehmood et al., 2023; Nicol et al., 2022; Paul et al., 2015; Pérez Rodríguez et al., 2021; Rasmussen et al., 2016; Reinhardt et al., 2022b; Sadeh et al., 2014; Saraff & Pepper, 2014; Shahwan et al., 2018; Shingleton et al., 2013; Silverman et al., 2018; Tan et al., 2014; Taş Torun et al., 2022; Thai et al., 2021; Vega et al., 2017; Victor & Klonsky, 2018; Wang et al., 2024; You et al., 2013, 2015; Zetterqvist et al., 2013). These motivations were found to be more salient to NSSI than other self-damaging behaviours, such as purging, binge drinking and binge eating (Robillard et al., 2022).

Notably, one study of refugee minors residing in Belgium found that the most common motivation was “practising suicide”, although this function often co-existed with other functions (Verroken et al., 2018). There were also studies showing that young people with a history of self-harm viewed interpersonal motivations as less relevant compared to peers without a self-harm history (Batejan et al., 2015; Doyle, 2017; Duarte et al., 2019) and parents of young people engaging in self-harm (Duarte et al., 2020a, 2020b).

### Correlates of Self-Harm Motivations

**Fig. 2:**
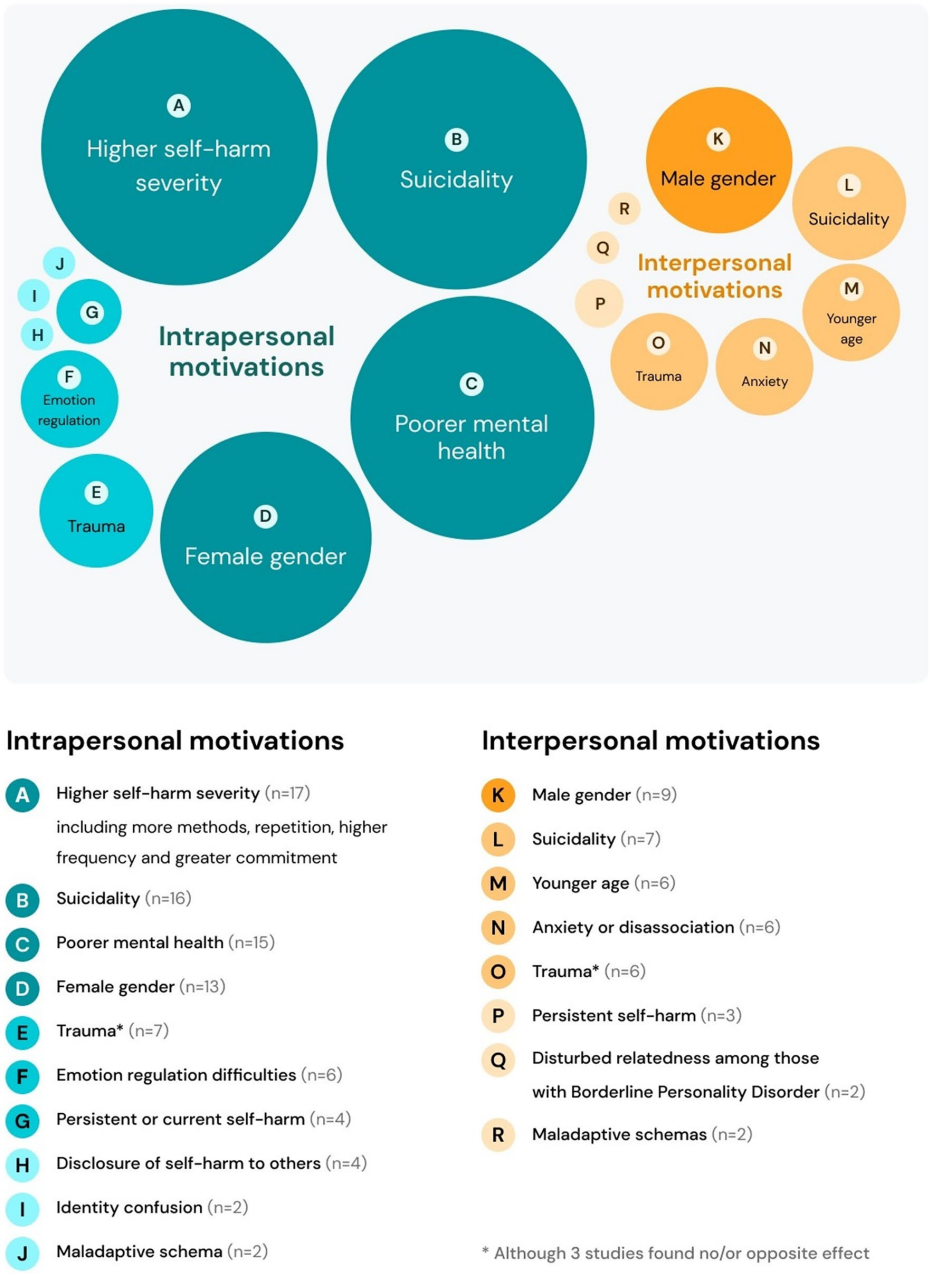
Correlates of intrapersonal (blue) and interpersonal (orange) motivations for self-harm among young people. The circles represent different correlates, with the size/shade of each circle corresponding with the number of studies finding a significant positive association between the correlate and the type of self-harm. Note that the figure does not include detail about the number of studies that find a non-significant or negative association between the given correlate and the type of self-harm motivation (Color figure online)

Figure 2 depicts key correlates of intrapersonal (orange) and interpersonal (blue) self-harm motivations. Larger circles/darker shades are indicative of a larger number of studies finding a significant positive association between the given correlate and the type of self-harm motivation. Note that the figure does not include detail about the number of studies that find a non-significant or negative association between the given correlate and the type of self-harm motivation. This information is instead summarised in written form below.

#### Gender

Gender was the most commonly investigated correlate, with a total of 24 studies investigating the association between gender and self-harm motivations. Nine studies found that intrapersonal motivations (as a composite) were more strongly endorsed among females compared to males (Faura-García et al., 2022; Ma & Su, 2023; Park et al., 2024; Reinhardt et al., 2021a, 2021b; Shen et al., 2023; Zetterqvist et al., 2013, 2014; Zhao et al., 2024). In terms of specific intrapersonal motivations, females were more likely than males to endorse affect regulation (DiCorcia et al., 2017; McManus et al., 2019; Reinhardt et al., 2022b) and anti-dissociation functions (Kostić et al., 2019) for self-harm, but not anti-suicide functions (DiCorcia et al., 2017). One study found that females were more likely to use self-harm “to feel something”, while males were more likely to use self-harm to manage anger (Andrei et al., 2024). Eight studies found no gender differences in intrapersonal motivations (Calvete Zumalde et al., 2015; Gholamrezaei et al., 2023; Güngördü & Ayaydin, 2024; Khutoryanskaya et al., 2023; Rasmussen et al., 2016; Reinhardt et al., 2022a; Selby et al., 2014; You et al., 2013). Only one study found that males were more likely to report intrapersonal motivations compared to females (Carvalho et al., 2023a).

In terms of interpersonal motivations, four studies found that sensation-seeking functions were more common in males compared to females (Khutoryanskaya et al., 2023; Reinhardt et al., 2021a, 2021b; Vega et al., 2017). Other interpersonal functions more commonly endorsed by males included positive social reinforcement (Shen et al., 2023), “wanting to frighten someone” (Rasmussen et al., 2016), “a cry for help” (Rasmussen et al., 2016), peer bonding (Güngördü & Ayaydin, 2024; Khutoryanskaya et al., 2023) and seeking attention from others (Zhao et al., 2024). Findings on gender differences regarding social influence functions were mixed (Kostić et al., 2019; You et al., 2013). Three studies (DiCorcia et al., 2017; Park et al., 2024; Zetterqvist et al., 2013) found no gender differences in interpersonal functions for self-harm.

#### Age

Eight studies examined the relationship between age and intrapersonal motivations for self-harm, with mixed findings. Four studies (DiCorcia et al., 2017; Nicol et al., 2022; Reinhardt et al., 2022b; Selby et al., 2014) found no association between age and intrapersonal motives for self-harm, while three other studies found a negative association between age and intrapersonal NSSI functions (Carvalho et al., 2023b; Reinhardt et al., 2021b; Shen et al., 2023). One study found that younger age was associated with self-punishment motivations (Khutoryanskaya et al., 2023).

There was more consistent evidence of an association between age and interpersonal functions, with six studies finding that interpersonal self-harm motives, including peer bonding, were negatively associated with current age or age of NSSI onset (Carvalho et al., 2023b; Gholamrezaei et al., 2023; Khutoryanskaya et al., 2023; Reinhardt et al., 2021a, 2021b, 2022b). One study found no association between age and any self-harm function (DiCorcia et al., 2017), while another study found a positive association between age and interpersonal functions (Nicol et al., 2022).

#### Characteristics of NSSI

Seven studies found that intrapersonal motivations were associated with moderate/severe NSSI as compared to mild NSSI (Batejan et al., 2015; Case et al., 2020; Gholamrezaei et al., 2023; Kharsati & Bhola, 2015; Luo et al., 2024; Nagy & Muehlenkamp, 2024; Reinhardt et al., 2022a), whereas two studies found that self-harm functions were not associated with NSSI severity (Abbasian et al., 2021; You et al., 2013). Endorsement of intrapersonal motivations for NSSI was also found to be positively associated with the number of applied self-harm methods, current self-harm, repetitive self-harm, self-harm frequency, pain and aloneness during self-harm, feelings of calm after self-harm, NSSI duration, addictiveness of NSSI, impulsive acts of self-harm and higher urge for and commitment to self-harm (Gardner et al., 2021; Gholamrezaei et al., 2023; Klonsky et al., 2015; Luo et al., 2024; Muehlenkamp et al., 2013; Rasmussen et al., 2016; Reinhardt et al., 2022a, 2022b; Saraff et al., 2015; Selby et al., 2014; Shen et al., 2023; Zetterqvist et al., 2014; Zhao et al., 2024). Additionally, intrapersonal motivations were found to explain variance in lifetime frequency and variety of NSSI above that afforded by interpersonal functions (Sadeh et al., 2014).

Four studies found that intrapersonal functions were more strongly endorsed among people with more persistent or current self-harm, as compared to those who had ceased self-harm (Gray et al., 2022; Pollak et al., 2020; Sadeh et al., 2014; Saraff & Pepper, 2014). This included a study that examined NSSI behaviours during an inpatient hospital stay among young people who had self-harmed (Pollak et al., 2020). A fifth study found that emotion regulation was the most common function among both those who had ceased, and those with persistent NSSI (Kiekens et al., 2017). A sixth study identified that use of NSSI for internal emotion regulation reasons was associated with lower motivation to stop self-injury (Kostic et al., 2024).

Regarding self-harm methods, one study found that self-cutting was more often used to avoid unwanted experiences (e.g. thoughts, memories and feelings), whereas self-hitting and scratching tended to be performed more to let out anger and frustration (You et al., 2015). Another study found that those who engaged in both physical self-harm and “digital self-harm” (self-harm involving posting or sharing harmful content about oneself online) endorsed self-punishment motives to a greater extent than those engaging in digital self-harm only (Wang et al., 2024).

Regarding the relationship between interpersonal functions and NSSI severity, three studies found that greater endorsement of interpersonal functions is associated with greater NSSI severity (Gholamrezaei et al., 2023; Luo et al., 2024; Thai et al., 2021), a fourth study found no relationship with NSSI severity (Reinhardt et al., 2022a), while a fifth study found that interpersonal functions were negatively associated with NSSI severity (Kharsati & Bhola, 2015). A study by Zhao et al (2024) found that the relationship between interpersonal functions and NSSI frequency was small. Studies found no association between interpersonal functions and repeat self-harm (with the exception of the function “I wanted to find out whether someone really loved me”), the number of applied self-harm methods, NSSI duration, NSSI frequency and total NSSI acts (Knorr et al., 2013; Rasmussen et al., 2016; Reinhardt et al., 2022b; Shen et al., 2023).

Regarding the relationship between interpersonal functions and the persistence of self-harm, two studies found that individuals who did not want to stop engaging in NSSI were more likely to engage in NSSI for sensation-seeking reasons (Gray et al., 2022). Similarly, a study by (Kiekens et al., 2017) found that sensation-seeking functions uniquely predicted persistent NSSI. However, Reinhardt et al. (2022b) found no association between interpersonal motives and current (vs. previous) self-harm.

The interpersonal functions, sensation-seeking and social influence, were found to be associated with specific methods of self-harm, including self-carving, self-burning and sex as a form of self-injury (Jonsson et al., 2019; Knorr et al., 2013).

#### Disclosure

Three studies examined the relationship between self-harm motives and disclosure of self-harm. Two studies found that endorsement of intrapersonal motivations for self-harm (including marking distress and anti-dissociation) was associated with greater likelihood of disclosure of self-harm to others (Ammerman et al., 2021; Mirichlis et al., 2022), whereas one study found no association between intrapersonal motivations for self-harm and disclosure (Armiento et al., 2014).

One study found that interpersonal motivations for self-harm were associated with disclosure of self-harm (Armiento et al., 2014), while another study found no such association (Ammerman et al., 2021).

#### Suicidality

There was consistent evidence of an association between intrapersonal self-harm functions and suicidality. Seven studies reported a positive association between intrapersonal functions (as a composite) and suicidal ideation or behaviour (DiCorcia et al., 2017; Park et al., 2022, 2024; Robinson et al., 2021; Shen et al., 2023; Victor & Klonsky, 2018; Wachter Morris & Wester, 2020). Specific intrapersonal functions found to be strongly associated with suicidality included anti-suicide, self-punishment and anti-dissociation functions, and automatic negative reinforcement functions more generally (Kostic et al., 2024; Kraus et al., 2020; Paul et al., 2015; Robinson et al., 2021; Roley-Roberts et al., 2017; Szewczuk-Bogusławska et al., 2021; Vergara et al., 2023; Victor et al., 2015; Wang et al., 2024). An ecological momentary assessment study found that on nearly all days during which suicidal ideation and NSSI co-occurred, adolescents reported to have engaged in NSSI to cope with their suicidal thoughts (Czyz et al., 2019). Notably, a longitudinal study found only weak evidence for an association between intrapersonal functions and future suicide attempts (Gardner et al., 2021), while a study conducted in Iran found no relationship between intrapersonal motivations and suicidality (Gholamrezaei et al., 2023).

Regarding the relationship between interpersonal functions and suicidality, five studies found that the motivations, establishing interpersonal boundaries, sensation-seeking and/or interpersonal influence, were associated with suicidality (Paul et al., 2015; Robinson et al., 2021; Vergara et al., 2023). Robinson et al. (2021) found that the interpersonal functions, peer bonding and toughness, were associated with less severe suicidality. Two studies (Park et al., 2024; Victor et al., 2015) found that while interpersonal functions were positively associated with suicidality, this relationship was stronger for intrapersonal functions. Victor et al. (2015) also found no association between suicide ideation and the following interpersonal functions: self-care, peer bonding, interpersonal influence and revenge.

Roley-Roberts et al. (2017) found that social negative reinforcement was associated with suicide ideation, but not suicide attempts, whereas Park et al. (2022) found no association between interpersonal functions and severity of suicidal ideation. Four studies found no association between interpersonal motivations for NSSI and suicidality (Gardner et al., 2021; Gholamrezaei et al., 2023; Kostic et al., 2024; Shen et al., 2023).

#### Mental Health

Intrapersonal motivations for self-harm were associated with depressive symptoms (DiCorcia et al., 2017; Gholamrezaei et al., 2023; Ong et al., 2017; Radziwiłłowicz & Lewandowska, 2017; Wang et al., 2024; Zetterqvist et al., 2014), dissociation (Radziwiłłowicz & Lewandowska, 2017; Zetterqvist et al., 2014), alexithymia (Ke et al., 2024), borderline personality disorder (Schmidt et al., 2023; Vega et al., 2017), poor body image (Radziwiłłowicz & Lewandowska, 2017), lower positive mental health (Reinhardt et al., 2022b), more dysregulated behaviours (e.g. alcohol use, impulsive spending, binge eating; Selby et al., 2014), anxiety (Gholamrezaei et al., 2023; Wang et al., 2024; Zhao et al., 2024) and the co-occurrence of two or more psychiatric disorders (Reinhardt et al., 2022b). Furthermore, intrapersonal motivations, including self-punishment and anti-disassociation, were associated with higher discrepancy (i.e. perceived difference between personal expectations and performance in meeting those expectations), self-critical rumination and lower self-esteem (Nagy & Muehlenkamp, 2024; Reinhardt et al., 2021b, 2022b). Among females specifically, intrapersonal motivations were found to be associated with higher loneliness, higher experiential avoidance and more pronounced levels of internalising and externalising mental illness symptoms (Rasmussen et al., 2016; Reinhardt et al., 2021b).

In terms of specific intrapersonal functions, use of self-harm for affect regulation reasons was associated with obsession-compulsion, anxiety, depressive symptoms, difficulty in identifying feelings and lack of clarity of emotional responses (DiCorcia et al., 2017; Taş Torun et al., 2022). Taş Torun et al. (2022) also found that the function, marking distress, was positively associated with paranoid ideation scores. Additionally, Selby et al. (2014) found that having automatic positive reinforcement functions for NSSI was associated with more dysregulated behaviours, including alcohol use, binge eating episodes and impulsive spending, but found no association with psychiatric disorders.

Interpersonal functions for self-harm were found to be positively associated with symptoms of anxiety and dissociation (Gholamrezaei et al., 2023; Ong et al., 2017; Radziwiłłowicz & Lewandowska, 2017; Wang et al., 2024; Zetterqvist et al., 2014; Zhao et al., 2024), whereas findings regarding the relationship between interpersonal functions for self-harm and depressive symptoms were mixed (Ong et al., 2017; Radziwiłłowicz & Lewandowska, 2017; Wang et al., 2024; Zetterqvist et al., 2014). The latter study also found a positive association between poor body image and the interpersonal boundaries function. One study found that interpersonal motivations were associated with lower loneliness and lower levels of internalising symptoms (Reinhardt et al., 2021b). Vega et al. (2017) found that the interpersonal motivations, interpersonal influence, self-care and sensation-seeking were more common for patients with borderline personality disorder compared to those without this diagnosis; however, Schmidt et al. (2023) found no association between interpersonal functions and borderline personality disorder. Reinhardt et al. (2022b) found no association between interpersonal functions and overall internalising and externalising symptoms.

Khutoryanskaya et al. (2023) found that self-harm motivations did not differ by mental health diagnoses (including behavioural disorders, depressive episodes, and eating disorders).

#### Emotion Reactivity

Overall, there was evidence to suggest that use of NSSI for intrapersonal reasons was positively associated with emotion regulation difficulties, with four studies finding a positive association between these variables (Carvalho et al., 2023a; Sadeh et al., 2014; Schmidt et al., 2023; Vega et al., 2017). Similarly, Christoforou et al. (2021) found that use of NSSI for intrapersonal reasons, including emotion regulation, self-punishment, anti-suicide and sensation-seeking functions, was associated with having considerable emotion difficulties. A fourth study found an association between emotional reactivity and automatic positive reinforcement functions, but not negative reinforcement functions (Kandsperger et al., 2022). Silverman et al. (2018) found no association between use of NSSI for emotion regulation reasons and emotional lability.

With respect to interpersonal functions and emotion regulation, a cluster analysis found that those with high emotion regulation difficulties and high tendency towards rumination reported higher social influence and sensation-seeking functions compared to clusters with less severe emotion regulation and rumination difficulties (Guérin-Marion et al., 2021). Furthermore, Christoforou et al. (2021) found that engaging in self-harm for peer bonding reasons was more relevant for those with passive-moderate emotion difficulties than those with no emotion difficulties, while (Gholamrezaei et al., 2023); Schmidt et al. (2023); (Wang et al., 2024; Zhao et al., 2024) found no association between interpersonal functions and emotion regulation difficulties.

#### Trauma

Four studies found positive associations between intrapersonal functions, and certain types of trauma, including emotional abuse, physical abuse, sexual abuse, neglect, parental antipathy and prolonged illness or handicap during upbringing (Balaji et al., 2023; Carvalho et al., 2023a; Kaess et al., 2013; Ke et al., 2024; Zetterqvist et al., 2014). Intrapersonal functions have also been found to be positively associated with the severity of post-traumatic stress symptoms (Ilieff & Hamza, 2023) and the severity of interpersonal trauma (Horowitz & Stermac, 2018). Two other studies found negative associations between certain intrapersonal functions and trauma (Radziwiłłowicz & Lewandowska, 2017; Taş Torun et al., 2022), while one study found no relationship between intrapersonal functions and intimate partner violence (Carranza et al., 2022).

In terms of the relationship between trauma and interpersonal functions, positive associations were found between use of NSSI for interpersonal reasons functions and sexual abuse (Bahali et al., 2024), neglect (Bahali et al., 2024; Carvalho et al., 2023b), parental antipathy (Carvalho et al., 2023b) and the severity of post-traumatic stress symptoms (Ilieff & Hamza, 2023). In terms of specific interpersonal functions, use of NSSI to establish interpersonal boundaries was found to be associated with both trauma severity (Horowitz & Stermac, 2018) and childhood physical abuse (Taş Torun et al., 2022), while use of NSSI for peer identification reasons was found to be associated with maternal physical abuse (Kaess et al., 2013). Notably, one study found a negative association between interpersonal functions and sexual abuse (Radziwiłłowicz & Lewandowska, 2017), while another study found a negative association between interpersonal functions and paternal apathy (Kaess et al., 2013). Zetterqvist et al. (2014) found no relationship between interpersonal functions and both emotional abuse and prolonged illness or handicap during upbringing.

#### Identity

Two studies found that intrapersonal functions negatively associated with identity synthesis (having a clear sense of identity) and positively associated with identity confusion (Gandhi et al., 2016; Luyckx et al., 2015). One of these studies found that interpersonal functions were not associated with sociotropy, autonomy or identity synthesis/confusion (Gandhi et al., 2016).

#### Social Functioning

One study found that intrapersonal functions were negatively associated with the size of one’s peer network; there was no association with interpersonal functions (Wachter Morris & Wester, 2020). Another study found that the interpersonal motive, establishing interpersonal boundaries, was not associated with problems in one’s interpersonal relationships (Silverman et al., 2018). Two studies found a positive association between interpersonal functions and disturbed relatedness among people with borderline personality disorder (Sadeh et al., 2014; Vega et al., 2017).

#### Maladaptive Schemas

Two studies examined the association between self-harm motivations and early maladaptive schemas. These studies found that intrapersonal functions of self-harm were associated with self-sacrifice, vulnerability to harm or illness, abandonment and entitlement schemas (Nicol et al., 2022; Shi et al., 2023). Interpersonal functions were associated with enmeshment, abandonment and insufficient self-control schemas (Nicol et al., 2022; Shi et al., 2023).

## Quality Assessment

The quality of included studies is summarised in Online Resource 3. Most studies adequately described their inclusion criteria (92.3%) and used appropriate statistical analyses (88.9%). Approximately, three quarters of included studies used a validated measure of self-harm (or ascertained self-harm using hospital records). A similar proportion of studies used a validated measure of self-harm motivations. Only one quarter of studies adequately described the study setting, with many studies not having reported on the period during which they recruited their sample.

## Discussion

The current review examined the motivations for self-harm among young people, aged 10–24. Unlike previous reviews, we identified correlates of different self-harm motivations, providing insight into differences between those acts of self-harm undertaken primarily for intrapersonal reasons as compared to those who self-harm for interpersonal reasons. Such insights provide a basis for future empirical research studies investigating self-harm in young people. Consistent with a previous review (Taylor et al., 2018), we found that the most common motivations for self-harm in this age group were intrapersonal (automatic motivations), with the most common being emotion regulation, anti-dissociation and self-punishment. Automatic negative reinforcement motivations were more common than positive reinforcement motivations. There were several correlates of intrapersonal self-harm motivations, including female gender, higher severity of NSSI, current, repetitive and persistent self-harm, suicidality, poorer mental health, trauma and poorer emotion regulation capacity. Interpersonal motives for self-harm were less prevalent compared to intrapersonal motives. There was evidence to suggest that interpersonal motives are correlated with male gender, younger age and some mental health difficulties (e.g. anxiety and dissociation).

The prevalence of intrapersonal motivations for self-harm reported in this review suggests that self-harm is typically used as an emotion regulation strategy among young people, particularly young women. This finding is consistent with existing research in this area (Taylor et al., 2018) and in line with the experiential avoidance model of self-harm, which purports that people engage in self-harm to avoid unwanted emotional experiences (Chapman et al., 2006). Qualitative studies have found that young people often perceive self-harm as an adaptive means of managing difficult thoughts or emotions (Hetrick et al., 2020; Miller et al., 2021; Mughal et al., 2023). Nevertheless, it remains important to reduce the rate of self-harm among young people, particularly given that we also found that those who engage in self-harm for intrapersonal reasons, including emotion regulation, are more likely to engage in more severe self-harm, alongside being at greater risk of suicidality and poorer mental health.

Indeed, it appears that there is a subset of individuals who engage in self-harm specifically as a way of managing suicidal thoughts. While anti-suicide motivations for self-harm were not as common as that of emotion regulation, anti-dissociation and self-punishment, it remains a common reason for self-harm among young people with a history of suicidality. Indeed, qualitative research has found that young people often view self-harm as a means of “survival” in response to suicidal thoughts or urges (Miller et al., 2021). Such findings suggest that interventions aimed at addressing self-harm in young people with a history of suicidality should not necessarily aim for complete cessation or reduction of self-harm, with some reporting concerns that doing so may increase distress (Cliffe & Stallard, 2023; Duperouzel & Fish, 2008). Rather, it has been suggested that interventions should address other factors, including general mental health and coping capacity (Cliffe & Stallard, 2023).

There was evidence of some gender differences in self-harm motivations, with several studies finding that females had greater endorsement of intrapersonal motivations compared to males. Contrastingly, there was evidence to demonstrate that males were more likely than females to engage in self-harm for sensation-seeking reasons (classified as an interpersonal motivation using the ISAS; Klonsky & Glenn, 2009). Gender differences in self-harm motivations may contribute to overall gender differences in the prevalence of self-harm. That is, the rate of self-harm might be higher among young females as this group may be more likely to use self-harm as a means of regulating their emotions. In contrast, males may be more likely to rely on other means of emotion regulation, or they may engage in indirect self-harm behaviours, such as substance use or high-risk activities, which are not captured as easily using existing self-harm assessment tools (Green & Jakupcak, 2016). It is therefore important to consider gender in theoretical models of self and harm and when designing interventions targeting self-harm. The current review suggests that what motivates a young person to engage in self-harm may be affected by factors such as gender, age and suicidality. While the results do not yet allow for the development of a comprehensive model of self-harm in this population, it does provide a knowledge base that guides the direction of future empirical studies. For instance, the review highlights correlates that have not been investigated by existing studies, such as sexual orientation and indigenous status. These variables have not been investigated despite research demonstrating those who identify as sexuality diverse, and those who identify as indigenous are at greater risk of self-harm (Dickson et al., 2019; McEvoy et al., 2023). The review also highlights correlates that require further research, such as self-harm methods and knowledge of peers engaging in self-harm. Such investigations may allow for the development of more tailored interventions. Further, the findings speak to potential subgroups of young people who engage in self-harm; namely how those who self-harm primarily for intrapersonal reasons differ from those who self-harm primarily for interpersonal reasons. It is also of note that there is empirical research demonstrating that young people often self-harm for multiple reasons (Sack et al., 2022; Shahwan et al., 2018); further research is required to enhance our understanding of how the function of self-harm can change according to time and context.

Although further empirical investigation is required, the results of the current review provide considerable insights into potential interventions for self-harm. Given that most young people who engage in self-harm do so for intrapersonal reasons, it is arguable that interventions that seek to reduce self-harm should aim to equip young people with alternative means of regulating their emotions (e.g. distress tolerance skills drawn from dialectical behavioural therapy; DBT). This may be particularly the case for young women and older adolescents. Notably, however, while there is some evidence to demonstrate that DBT may reduce the repetition of self-harm in young people, additional research is required to establish its efficacy (Witt et al., 2021). Considerations should also be directed towards the type of delivery for these interventions. For example, while schools may be an appropriate setting to deliver interventions for self-harm, there is evidence suggesting that targeted approaches that are aimed at those who are at greater risk may be more effective than universal school-based mental health interventions (Werner-Seidler et al., 2021; Zbukvic et al., 2024). Because there are individual differences in self-harm motivations among young people, a targeted or individually orientated approach might be most effective. Online-based interventions for self-harm have become increasingly available, but further evidence of their capacity to reduce self-harm is required (Arshad et al., 2020).

Given the complexity of self-harm in terms of the role of genetic, biological, psychiatric, sociocultural risk factors, the reduction or prevention of self-harm requires a multifaceted approach. Beyond providing young people with alternative ways of regulating their emotions, it is of interest to better understand the circumstances or stressors that are leading to distress and increased emotion dysregulation among young people. Existing reviews have suggested that common precipitants for self-harm include problems with family or friends (Rahman et al., 2021). The current review found links between trauma and use of self-harm for intrapersonal reasons. Whether precipitants for self-harm in young people have changed in type or prevalence over time is unclear. For instance, it is possible that self-harm rates are increasing due to increasing uncertainty faced by young people in response to the current political, economic and environment issues. It is also possible that social media plays a role in exposing young people to self-harm content (Susi et al., 2023), although evidence that social media has driven the recent rise in self-harm is weak (Moran et al., 2024) and needs further investigation. Longitudinal and qualitative study designs may be useful in providing insight into why self-harm rates have risen over the past decade.

There are several limitations of the current review. Firstly, this review did not include qualitative studies, which may have provided richer information about the experience of self-harm among young people. Several existing systematic reviews have examined self-harm motivations using qualitative studies (Edmondson et al., 2016; Stänicke et al., 2018) and we plan to provide a synthesis of qualitative studies to complement the current review, using the papers identified in the current search. This synthesis will involve qualitative analysis of these studies and, together with the current review, will provide further insights into the drivers of self-harm in young people. Furthermore, only 29% of included studies included a representative sample, limiting the generalisability of our findings. Studies predominantly used community or school-based samples of young people, with few studies examining samples of young people who had been hospitalised for self-harm, and only two studies examined the association between self-harm motivations and methods. Given the increase in rates of self-harm hospitalisations among young people, it would be of interest to determine whether those who are hospitalised for self-harm differ in their motivations compared to those who are not hospitalised, as interventions likely need to be tailored according to self-harm severity.

Taken together, the findings of this review indicate that young people predominantly self-harm to regulate their emotions. Given that self-harm for intrapersonal reasons is associated with increased severity of NSSI, suicidality and poor mental health, it is important that steps are taken to reduce or prevent self-harm among young people. Interventions for self-harm require a multifaceted approach that not only provides young people with alternate ways of regulating their emotions, but also targets risk factors that contribute to self-harm.

## Supplementary Information

Below is the link to the electronic supplementary material.

Supplementary file1 (DOCX 25 KB)

Supplementary file2 (DOCX 26 KB)

Supplementary file3 (DOCX 48 KB)
