# Supplementary material for: Motivations for Self-Harm in Young People and Their Correlates: A Systematic Review
Source: Clin Child Fam Psychol Rev. 2025 Jan 29;28(1):171–208. doi: 10.1007/s10567-024-00511-5 (PMC11885408; doi:10.1007/s10567-024-00511-5)
Supplement: Supplementary file 1 — Supplementary file1 (DOCX 25 KB) [file 10567_2024_511_MOESM1_ESM.docx]

**Online Resource 1.** Search strategy

| **Database** | **Population AND** | **Exposure AND** | **Cause/**  **Motivation/**  **Process** |
| --- | --- | --- | --- |
| **PsycINFO via Ovid**  Limits =  English language,  Human, peer reviewed journal, past 10 years  **N = 2903**  Original search date: 23/3/23  Updated search date: 10/9/24 | (child* or adolescen* or youth or young people or young adult* or teen* or student* or young person).ti,ab. | (suicide attempt* or self-harm or self harm or self-poison* or self poison* or self-injur* or self injur* or self-cut* or self cut* or self-mutilat* or self mutilat* or parasuicid* or suicidal behav* or DSH or suicidal attempt* or attempted suicide* or suicidal act*). ti,ab. | (motiv* or planned or planning or intention or intentions or intent or premeditate* or opportunistic or impuls* or reason* or explanation* or precipita* or caus* or circumstance* or stressor* or choice* or chose or choos* or function or functions or meaning* or factors or explain*). ti,ab. |
| **Embase via Ovid**  Limits = English language,  Human, remove preprint records, not conference abstracts, past 10 years  **N = 4561**  Original search date: 23/3/23  Updated search date: 10/9/24 | (child* or adolescen* or youth or young people or young adult* or teen* or student* or young person).ti,ab. | (suicide attempt* or self-harm or self harm or self-poison* or self poison* or self-injur* or self injur* or self-cut* or self cut* or self-mutilat* or self mutilat* or parasuicid* or suicidal behav* or DSH or suicidal attempt* or attempted suicide* or suicidal act*). ti,ab. | (motiv* or planned or planning or intention or intentions or intent or premeditate* or opportunistic or impuls* or reason* or explanation* or precipita* or caus* or circumstance* or stressor* or choice* or chose or choos* or function or functions or meaning* or factors or explain*). ti,ab. |
| **Medline**  **via Ovid**  Filters =  English,  Human, 10 years  **N = 3479**    Original search date: 23/3/23  Updated search date: 10/9/24 | child*[Title/Abstract] OR adolescen*[Title/Abstract] OR youth[Title/Abstract] OR "young people"[Title/Abstract] OR "young adult*"[Title/Abstract] OR teen*[Title/Abstract] OR student*[Title/Abstract] OR "young person"[Title/Abstract] | "suicide attempt*"[Title/Abstract] OR self-harm[Title/Abstract] OR "self harm"[Title/Abstract] OR self-poison*[Title/Abstract] OR "self poison*"[Title/Abstract] OR self-injur*[Title/Abstract] OR "self injur*"[Title/Abstract] OR self-cut*[Title/Abstract] OR "self cut*"[Title/Abstract] OR self-mutilat*[Title/Abstract] OR "self mutilat*"[Title/Abstract] OR parasuicid*[Title/Abstract] OR "suicidal behav*"[Title/Abstract] OR DSH[Title/Abstract] OR "suicidal attempt*"[Title/Abstract] OR "attempted suicide*"[Title/Abstract] OR "suicidal act*"[Title/Abstract] | motiv*[Title/Abstract] OR planned[Title/Abstract] OR planning[Title/Abstract] OR intention[Title/Abstract] OR intentions[Title/Abstract] OR intent[Title/Abstract]OR premeditate*[Title/Abstract] OR opportunistic[Title/Abstract] OR impuls*[Title/Abstract] OR reason*[Title/Abstract] OR explanation*[Title/Abstract] OR precipita*[Title/Abstract] OR caus*[Title/Abstract] OR circumstance*[Title/Abstract] OR stressor*[Title/Abstract] OR choice*[Title/Abstract] OR chose[Title/Abstract] OR choos*[Title/Abstract] OR function[Title/Abstract] OR functions[Title/Abstract] OR meaning*[Title/Abstract] OR factors[Title/Abstract] OR explain*[Title/Abstract] |
