# Supplementary material for: Motivations for Self-Harm in Young People and Their Correlates: A Systematic Review
Source: Clin Child Fam Psychol Rev. 2025 Jan 29;28(1):171–208. doi: 10.1007/s10567-024-00511-5 (PMC11885408; doi:10.1007/s10567-024-00511-5)
Supplement: Supplementary file 3 — Supplementary file3 (DOCX 48 KB) [file 10567_2024_511_MOESM3_ESM.docx]

**Online Resource 3.** Quality of included studies.

|  | **Item 1. Inclusion criteria** | **Item 2. Study setting** | **Item 3. Exposure variables** | **Item 4. Outcome measure** | **Item 5. Appropriate analysis** |
| --- | --- | --- | --- | --- | --- |
| Abbasian et al. (2021) | + | + | + | + | + |
| Ammerman et al. (2021) | + | + | + | + | + |
| Andrei et al. (2024) | + | + | + | + | - |
| Andrewes et al. (2017) | + | / | + | / | + |
| Armiento et al. (2014) | + | / | + | + | + |
| Babcock Fenerci et al. (2022) | + | / | + | + | + |
| Bahali et al. (2024) | + | / | + | + | + |
| Barreto Carvalho et al. (2017) | + | / | + | + | + |
| Batejan et al. (2015) | + | / | + | + | / |
| Bentley et al. (2015) | + | / | + | + | + |
| Braga & Gonçalves (2014) | / | / | + | + | + |
| Brausch et al. (2016) | + | / | + | + | + |
| Brausch & Muehlenkamp (2018) | / | / | / | / | + |
| Calvete et al. (2015) | / | / | / | / | + |
| Carranza et al. (2022) | + | / | + | + | + |
| Carvalho et al. (2023a) | + | / | + | + | + |
| Carvalho et al. (2023b) | / | / | + | + | + |
| Case et al. (2020) | + | / | + | + | + |
| Christoforou et al. (2021) | + | / | + | + | + |
| Coppersmith et al. (2021) | + | / | / | - | + |
| Costa et al. (2021) | + | + | + | + | + |
| Czyz et al. (2019) | + | / | + | - | + |
| Czyz et al. (2021) | + | / | / | / | + |
| DiCorcia et al. (2017) | + | / | / | / | + |
| Dixon-Gordon et al. (2022) | + | / | + | + | + |
| Doyle (2017) | + | / | + | + | + |
| Doyle et al. (2017) | + | / | / | / | + |
| Duarte et al. (2019) | + | / | + | + | + |
| Duarte et al. (2020) | + | / | + | + | - |
| Faura-García et al. (2022) | + | + | + | + | + |
| Gandhi et al. (2016) | + | / | / | + | + |
| Gandhi et al. (2021) | + | / | - | / | + |
| García-Nieto et al. (2015) | + | + | + | + | + |
| Gardner et al. (2021) | + | + | - | - | + |
| Gatta et al. (2022) | + | + | + | / | + |
| Gholamrezaei et al. (2023) | + | / | / | / | / |
| Goddard et al. (2021) | + | / | + | + | + |
| Gray et al. (2022) | + | / | + | + | + |
| Groschwitz et al. (2015) | + | + | + | + | + |
| Guan et al. (2024) | + | + | + | + | + |
| Guérin-Marion et al. (2021) | + | + | + | + | + |
| Gungordu & Ayaydin (2024) | + | + | + | + | / |
| Hamza et al. (2014) | + | / | + | + | + |
| Hettiarachchi et al. (2018) | + | / | - | - | + |
| Horowitz & Stermac (2018) | + | / | + | + | + |
| Idig-Camuroglu & Gölge  (2018) | + | / | + | + | / |
| Ilieff & Hamza (2023) | + | / | / | / | + |
| Jiang et al. (2022) | + | / | + | + | + |
| Jonsson et al. (2019) | + | / | + | + | + |
| Kaess et al. (2013) | + | + | + | + | + |
| Kandsperger et al. (2022) | + | + | + | + | + |
| Khutoryanskaya et al.  (2023) | + | / | + | + | / |
| Kharsati & Bhola (2015) | + | / | + | + | + |
| Kiekens et al. (2017) | + | / | + | + | + |
| Klonsky et al. (2015) | + | / | + | + | + |
| Knorr et al. (2013) | + | / | + | + | + |
| Kostić et al. (2019) | + | + | + | + | + |
| Kostić et al. (2024) | + | + | + | + | + |
| Kraus et al. (2020) | + | / | + | + | + |
| Lee (2016) | + | / | / | + | + |
| Luo et al. (2024) | + | / | + | + | + |
| Luyckx et al. (2015) | + | / | + | + | + |
| Ma et al. (2023) | + | / | + | + | + |
| Mahtani et al. (2018) | + | / | + | + | + |
| McManus et al. (2019) | + | + | - | - | + |
| Mehmood et al. (2023) | + | + | + | + | / |
| Mirichlis et al. (2022) | / | / | + | + | + |
| Muehlenkamp et al. (2013) | + | / | + | + | + |
| Nagy & Muehlenkamp (2024) | + | / | + | + | + |
| Nicol et al. (2022) | + | / | / | / | + |
| Ong et al. (2017) | + | / | + | + | + |
| Park et al. (2022) | + | / | + | + | + |
| Park & Ammerman (2024) | + | / | + | + | + |
| Paul et al. (2015) | + | / | / | / | + |
| Pérez et al. (2021) | + | + | + | + | + |
| Peters et al. (2019) | + | / | + | + | + |
| Piarulli et al. (2023) | + | + | + | + | + |
| Pollak et al. (2020) | + | / | + | + | + |
| Radziwiłłowicz & Lewandowska (2017) | - | - | + | + | / |
| Rasmussen et al. (2016) | + | / | / | / | + |
| Reinhardt et al. (2021a) | + | + | + | + | + |
| Reinhardt et al. (2021b) | + | + | + | + | + |
| Reinhardt et al. (2022a) | + | / | + | + | + |
| Reinhardt et al. (2022b) | + | + | + | + | + |
| Robillard et al. (2022) | + | + | - | / | + |
| Robinson et al. (2021) | + | / | + | + | + |
| Rodav et al. (2014) | + | / | + | + | + |
| Roley-Roberts et al. (2017) | + | / | + | + | + |
| Sadeh et al. (2014) | + | + | + | + | + |
| Saraff & Pepper (2014) | / | / | + | + | + |
| Saraff et al. (2015) | + | / | + | + | + |
| Schmidt et al. (2023) | + | / | + | + | + |
| Selby et al. (2014) | + | / | + | - | + |
| Shahwan et al. (2018) | + | + | + | + | + |
| Shen et al. (2023) | + | + | + | + | + |
| Shi et al. (2023) | / | + | + | + | + |
| Shingleton et al. (2013) | + | / | / | - | / |
| Silverman et al. (2018) | + | / | + | + | + |
| Szewczuk-Bogusławska et al. (2021) | + | / | - | + | + |
| Tan et al. (2014) | + | / | + | + | / |
| Taş Torun et al. (2022) | + | / | + | + | / |
| Thai et al. (2021) | + | / | + | + | + |
| Vega et al. (2017) | + | / | / | / | + |
| Vergara et al. (2023) | + | / | + | + | + |
| Verroken et al. (2018) | + | + | / | / | + |
| Victor et al. (2015) | + | / | + | + | + |
| Victor & Klonsky (2018) | + | / | - | + | + |
| Wachter Morris & Wester (2020) | + | / | / | + | + |
| Wang et al. (2022) | + | + | + | + | + |
| Wang et al. (2024) | + | + | / | / | + |
| Westers et al. (2014) | / | / | + | + | + |
| You et al. (2013) | + | / | - | / | + |
| You et al. (2015) | + | / | + | + | / |
| Zetterqvist et al. (2013) | + | + | + | + | + |
| Zetterqvist et al. (2014) | + | / | + | + | + |
| Zhang et al. (2019) | + | / | + | + | + |
| Zhao et al. (2024) | + | + | + | + | + |
| % Adequate | 92.3% | 28.2% | 77.8% | 78.6% | 88.9% |
| % Partial | 6.8% | 70.9% | 15.4% | 15.4% | 9.4% |
| % Poor/unclear | 0.9% | 0.9% | 6.8% | 6.0% | 1.7% |

*Note.* N = 117. + Adequate; / Partial; - Poor/unclear.
